# A comparative analysis of extracellular vesicles (EVs) from human and feline plasma

**DOI:** 10.1038/s41598-022-14211-z

**Published:** 2022-06-27

**Authors:** Jane Howard, Kieran Wynne, Evelin Moldenhauer, Paul Clarke, Ciaran Maguire, Stephanie Bollard, Xiaofei Yin, Lorraine Brennan, Louise Mooney, Stephen Fitzsimons, Melinda Halasz, Ester Rani Aluri, Dermot F. Brougham, Walter Kolch, Róisín M. Dwyer, Shirley Potter, Pamela Kelly, Amanda McCann

**Affiliations:** 1 UCD School of Medicine, College of Health and Agricultural Sciences, University College Dublin, Belfield, Dublin 4, Ireland; 2 UCD Conway Institute of Biomolecular and Biomedical Research, University College Dublin, Belfield, Dublin 4, Ireland; 3 Systems Biology Ireland, University College Dublin, Belfield, Dublin 4, Ireland; 4 Postnova Analytics GmbH, Rankinestrasse 1, 86899 Landsberg, Germany; 5 Postnova Analytics UK Ltd, Malvern Hills Science Park, Malvern, WR14 3SZ Worcestershire UK; 6 Particular Sciences Ltd, Unit 2 Birch House, Rosemount Business Park, Ballycoolin, Dublin 11, Ireland; 7 Department of Plastic & Reconstructive Surgery, Mater Misericordiae University Hospital, Dublin 7, Ireland; 8 UCD School of Agriculture and Food Science, University College Dublin, Belfield, Dublin 4, Ireland; 9 College of Health and Agricultural Sciences, UCD School of Veterinary Medicine, University College Dublin, Belfield, Dublin 4, Ireland; 10 Diabetes Complications Research Centre, School of Biomolecular and Biomedical Sciences, UCD Conway Institute, Belfield, Dublin 4, Ireland; 11 UCD School of Chemistry, University College Dublin, Belfield, Dublin 4, Ireland; 12 Discipline of Surgery, Lambe Institute for Translational Research, National University of Ireland Galway, Galway, H91 V4AY Ireland

**Keywords:** Biological techniques, Health care, Molecular medicine, Chemical biology

## Abstract

Extracellular vesicles (EVs) are nanoparticles found in all biological fluids, capable of transporting biological material around the body. Extensive research into the physiological role of EVs has led to the development of the Minimal Information for Studies of Extracellular Vesicles (MISEV) framework in 2018. This framework guides the standardisation of protocols in the EV field. To date, the focus has been on EVs of human origin. As comparative medicine progresses, there has been a drive to study similarities between diseases in humans and animals. To successfully research EVs in felines, we must validate the application of the MISEV guidelines in this group. EVs were isolated from the plasma of healthy humans and felines. EV characterisation was carried out according to the MISEV guidelines. Human and feline plasma showed a similar concentration of EVs, comparable expression of known EV markers and analogous particle to protein ratios. Mass spectrometry analyses showed that the proteomic signature of EVs from humans and felines were similar. Asymmetrical flow field flow fractionation, showed two distinct subpopulations of EVs isolated from human plasma, whereas only one subpopulation was isolated from feline plasma. Metabolomic profiling showed similar profiles for humans and felines. In conclusion, isolation, and characterisation of EVs from humans and felines show that MISEV2018 guidelines may also be applied to felines. Potential comparative medicine studies of EVs may provide a model for studying naturally occurring diseases in both humans and felines.

## Introduction

Extracellular vesicles (EVs) are naturally released particles that cannot self-replicate, delimited by a lipid bilayer. The concept of EVs was first coined by Rose Johnstone^1^ in 1989, and since then, significant efforts have followed to develop the EV field^2^. While various subtypes of EVs have been classified based on physical properties such as exomere, exo-small (exo-S) and exo-large (exo-L)^3^, recent studies have shown that it is virtually impossible to distinguish between these populations when isolating EV populations^4^. For this reason, when isolating nanoparticles from biological fluids, the term ‘*extracellular vesicle’* is used^5^. EVs are highly representative of their cells of origin. Their formation involves a unique regulatory process, which likely determines their biological function once secreted from the cell into the extracellular space. Their function is also likely influenced by their composition. They contain biomolecules such as DNA, RNA, lipids and metabolites. The specific roles of EVs include the removal of excess cell constituents, the maintenance of cellular homeostasis, and the facilitation of cellular communication. Additional research has also demonstrated the ability of EVs to deliver therapeutics in vivo^6^.

The international society for extracellular vesicles (ISEV) was established in 2011, followed by the creation of a peer reviewed open access Journal of Extracellular Vesicles in 2012. The ISEV published guidelines in 2014 entitled “Minimal experimental requirements for definition of extracellular vesicles and their functions: a position statement from the International Society for Extracellular Vesicles” (MISEV 2014)^2^, implemented to encourage rigor and standardisation when attributing a particular biological cargo or function to EVs. The primary goal of the MISEV recommendations, was to sensitise researchers, as well as journals and reviewers to experimental reporting requirements specific to the EV field. The ISEV highlighted the need to consider these issues when making strong conclusions about EVs. Based on collective developments, and knowledge from EV studies, the guidelines were further updated in 2018^5^. The MISEV 2018 authors hoped to further the promise of EVs as biomarkers or for therapeutic applications even in the face of skepticism by some scientists outside the field^5^. The MISEV 2018 guidelines include tables, protocols, and checklists to follow to document specific EV-associated functional activities. One of the most critical aspects of the MISEV guidelines to date, is their focus on EVs from human biological fluids or cell lines^5^. While human studies are a useful starting point, EV research has expanded to include other studies in veterinary medicine and marine biology^7^.

Comparative medicine has been described as the integration of naturally occurring diseases seen in veterinary patients into more general studies of biology and therapy. Most biological research studies include murine studies. However, mice have a short lifespan, and many lack an intact immune system, which makes studies very difficult. Studying naturally occurring diseases in the veterinary setting is of benefit to both human and veterinary patient cohorts, and bridges the gap between fundamental diagnostics, therapeutics, and clinical trials for both. Although several veterinary species including the cat^8^, horse^9^, pig^10^ and ferret^11^ have demonstrated comparative investigative potential, the majority of research investigations so far have focused on the dog^8^. The role of EVs in pathologies such as ageing^12^, cancer^13^, infectious diseases^14^, and obesity^15^ has widespread interest in human disease. However, the EV field has received less attention in the veterinary setting.

Domestic cats share many diseases with humans. Like humans, cats living in domestic homes are exposed to similar lifestyle risk factors which may influence the development of diseases. In domestic cats, passive smoking has been shown to increase the risk of developing malignant lymphoma^16^, while obesity increases the risk of diabetes mellitus development^17^. Therefore, EV studies of domestic cats may contain integral information to unravel the pathogenesis of these comparative diseases. To date, EV studies on felines have been limited to cell line derived EVs^7^ and there has been no research focused on EVs isolated from feline biological fluids.

Liquid biopsy, or fluid biopsy, refers to the sampling of non-solid biological tissue, mainly in the form of blood, plasma, or serum. Although routinely carried out in human hospitals and veterinary practices, the isolation of EVs or other biologically relevant nanoparticles is still somewhat uncommon^18^. EVs are reflections of their cells of origin. Therefore, their isolation from liquid biopsies harbours a wealth of undiscovered biological information^19^. For feline cohorts there have been no published studies detailing the isolation of EVs from feline liquid biopsies. As the applicability of liquid biopsy studies in felines has not yet been explored, it is important to investigate whether it is possible to isolate and characterise EVs from feline plasma using the same techniques as we would with human plasma. Therefore, before studying EVs in the context of feline diseases, we must first investigate the ‘normal’ or non-diseased characteristics of EVs isolated from feline plasma.

The first step in researching EVs from felines, is to establish whether the guidelines from the MISEV 2018 position paper^5^ are applicable to feline studies, and whether they can be strictly followed for this patient cohort. Previous opinion has deemed the MISEV guidelines as being too restrictive or too strong an imposition on the field, raising the question about whether they are in fact stifling EV research in the veterinary field^5^. MISEV 2018 also states that they encourage the development of the MISEV guidelines using the principles of the document as a guide.

**Figure 1:**
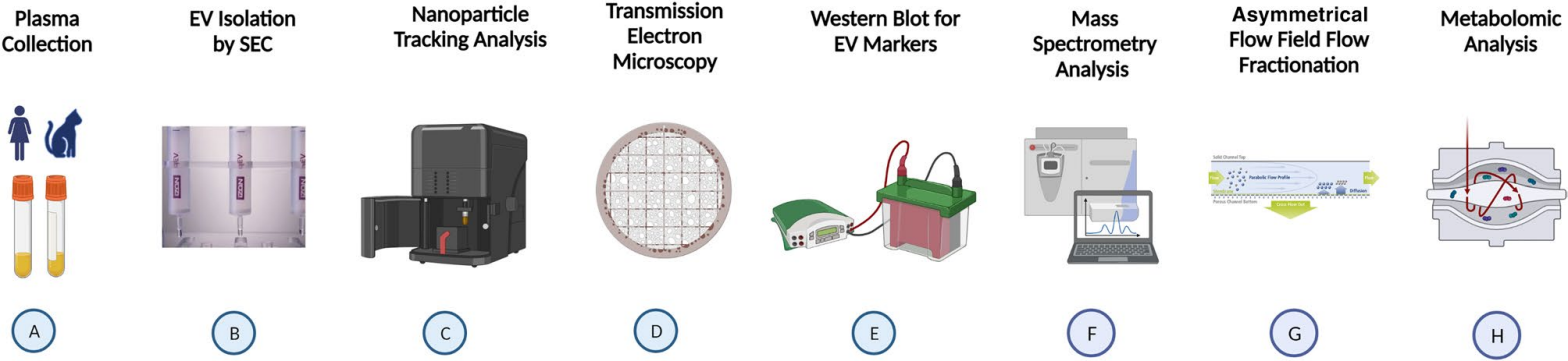
Schematic workflow detailing the step-by-step methodology from EV isolation to characterisation and further EV analysis. EVs were isolated from human and feline plasma by size exclusion chromatography (SEC), characterisation by nanoparticle tracking analysis (NTA), transmission electron microscopy (TEM) and western blot, according to the MISEV 2018 guidelines. Further analysis was carried out by mass spectrometry (LC–MS/MS), asymmetrical flow field flow fractionation (AF4) and metabolomics. (Image was created with BioRender.com).

To investigate the applicability of EV studies as biomarkers of disease in feline patients, we evaluated the similarity and differences between EVs isolated from feline plasma and EVs isolated from human plasma. In the current study, we were guided by MISEV 2018, as we isolated EVs from the plasma of healthy humans and felines by size exclusion chromatography (SEC). We investigated the concentration of particles, protein content and shape of EVs from feline plasma, and compared them to EVs from human plasma. Harvested EVs from humans and felines were characterised according to the MISEV 2018 guidelines^5^ by nanoparticle tracking (NTA), transmission electron microscopy (TEM) and western blot analyses. EVs from both groups were then examined by mass spectrometry (MS) and asymmetric field flow fractionation (AF4). Further analysis of the metabolomic content of these EVs was also carried out (Fig. 1).

This study has assessed the suitability of the MISEV guidelines^5^ to characterise EVs from human and feline plasma and investigated the similarities and differences between human and feline plasma derived EVs. The overarching objective is to determine the suitability of human and feline plasma EVs for future comparative studies. Before translational investigations into similar diseases in different species are undertaken, it is imperative that the similarities and differences in EVs from a non-disease cohort is established.

## Methods

### Sample collection and preparation

Human whole blood donations were obtained from the Irish Blood Transfusion Service, which provides de-identified blood components, and bi-products of the donation process, *pro bono* to academic researchers following approval (Irish Blood Transfusion Service Approval Number: 001-03-19). All experiments were performed in accordance with the UCD Conway Institute Guidelines, and in compliance with international laws. All volunteers gave informed, written consent.

Human blood samples were spun at 1900×*g* for 15 min to separate out the plasma. Human plasma was then spun at 2500×*g* for 10 min to remove platelets. The plasma from 3 humans, was then pooled, and 500 µl aliquots were stored at − 80 °C for subsequent EV isolation.

Plasma samples taken as part of the routine veterinary care from the feline cohort, were accessed only, after all clinical diagnostic tests were carried out. Owner consent was obtained, and all experiments were performed in accordance with the protocol approved by the Animal Research Ethics Committee, UCD (Approval ID: AREC-E-21-32-Kelly). Residual plasma samples from felines who had blood withdrawals as part of their normal veterinary care were accessed, only after all diagnostic tests requested by their treating veterinarian were complete. Supplementary information, Sect. 1 outlines the biochemistry parameter values available including reference ranges for these samples. Feline blood samples were spun at 1900×*g* for 15 min to separate out the plasma. Feline plasma was subsequently spun at 2500×*g* for 10 min to remove platelets. Plasma from 20 felines was then pooled, and 500 µl aliquots were stored at − 80 °C for subsequent EV isolation. For both humans and felines, leftover plasma was used following routine diagnostic testing or blood donation. In both cases plasma isolation has been optimised for clinical diagnostic procedures in human and veterinary clinics. Therefore, plasma from humans and felines is of similar quality. Additionally, by using leftover plasma, we are confident that EV studies using a liquid biopsy approach may be carried out on plasma samples in both human and veterinary clinics in the future.

We note the importance of providing enough information to demonstrate the reliability and robustness of the findings allowing others to replicate and build upon our study. However in this instance, the ARRIVE guidelines (e.g. animal care and monitoring, housing and husbandry) are not appropriate for this study, as it did not involve an *in-vivo* experiment. Rather, these animals are pets being cared for by their owners. All experiments were performed in accordance with relevant guidelines and regulations.

### iZon isolation of EVs

EVs were isolated from plasma by size exclusion chromatography (SEC), using IZON qEV original columns (iZon Science). The columns were removed from 4 °C, and the 20% ethanol storage removed. Isolations were performed identically for both humans and felines, and fractions collected using an automated fraction collector (iZon Science). The column was flushed with 10 ml of PBS, prior to sample collection. To isolate EVs from human plasma, a 500 µl aliquot of previously pooled plasma was removed from − 80 °C storage and thawed quickly. For each isolation, 500 µl of plasma was added to the column and fractions were collected immediately. Fractions 1–12 each containing 500 µl of elute were collected and buffer was added to the top of the column. Columns were washed and re-used according to the manufacturer’s instructions. According to the manufacturer’s guidelines, fractions 7–10 were pooled to limit the number of freeze thaw cycles and subsequently aliquoted for downstream analysis and stored at − 80 °C.

Similarly, to isolate EVs from feline plasma, a 500 µl aliquot of previously pooled plasma was removed from − 80 °C storage and thawed quickly. For each isolation, 500 µl of plasma was added to the column and fractions were collected immediately. Separate columns were used for human and feline isolations, to avoid any cross contamination. Fractions 1–12 each containing 500 µl of elute were collected and buffer was added to the top of the column. Columns were washed and re-used according to the manufacturer’s instructions. According to the manufacturer’s guidelines, fractions 7–10 were pooled to limit the number of freeze thaw cycles and subsequently aliquoted and stored at − 80 °C for downstream analysis.

### Nanoparticle tracking analysis

Nanoparticle tracking analysis (NTA) using a NanoSight NS300 system (Malvern Technologies, Malvern, UK), configured with a 488 mm later and a high sensitivity CMOS camera was used to determine the size distribution and concentration of EVs from fractions 1–12 following isolation by SEC. Pooled fractions 7–10 were also analysed. Samples were diluted in fresh filtered PBS (1:25 dilution) and loaded into the sample chamber. Samples were analysed under constant flow conditions (flow rate = 50) at 25 °C, camera level 12 and screen gain 2, for each run. Five × 1 min videos were captured for each sample. Human and feline EVs were analysed in duplicate. Data was analysed using NTA 4.1 software, with a detection threshold of 10 and bin size of 2.

### EV protein isolation and quantification

To establish the protein concentration of each EV fraction isolated by SEC, 50 µl of each EV Fraction 1–12 were collected and lysed using 10 µl RIPA Buffer [50 mM Tris HCL pH 7.4, 150 mM NaCl, 1% NP40, 1% EDTA, 1 mM phenylmethylsulfonyl, 1% protease inhibitor and 1% phosphatase inhibitor]. Samples were left on ice for 15 min, followed by centrifugation at 10,000 × g for 20 min at 4 °C. Samples were sonicated for three minutes. Protein quantification was carried out using the Pierce BCA Protein Assay Kit (ThermoFisher Scientific: 23,225) in duplicate according to the manufacturer’s instructions. A 25 µl aliquot of EV lysate was added to a 96-well microplate, followed by 200 µl of BCA reagent (ThermoFisher, USA). The plate was subsequently incubated in light exclusion for 30 min at 37 °C and absorbance was measured at 560 nm. The protein concentration was determined from a BSA standard curve.

### Transmission electron microscopy (TEM)

Pooled EVs from human fractions 7–10 and feline fractions 7–10 were concentrated. 10 µl of isolated EVs were placed on a formvar carbon-coated copper EM grid for 60 min. Vesicle coated grids were washed three times with PBS then fixed using 2.5% glutaraldehyde for 10 min. After washing in distilled water, the grids were stained in 2% uranyl acetate for 15 min and embedded by methyl cellulose-UA for 10 min on ice. Excess cellulose was removed, and samples were air dried for 20 min. Transmission electron microscopy was performed using a FEI Tecnai 120 microscope, operating at an accelerating voltage of 120 kV. Images were taken of the entire field, at 87000X and individual vesicles in that given field were observed at 16500X.

### SDS polyacrylamide gel electrophoresis (SDS-PAGE) and western blot analysis

The protein content of human and feline EVs were analysed in triplicate. For each sample, fractions 7–10 were combined and concentrated. A protein concentration of 4 ng was mixed with 5 µl 1 × blue loading buffer (NewEngland BioLabs, B7703S) and 1.25 M DTT and heated to 95 °C for 5 min. Proteins were run on an 8–12% SDS NuPAGE Bis–Tris gel (Invitrogen, NP0326BOX). These gels were run in NuPAGE MOPS SDS running buffer at 200 V for 42 min. Resolved proteins were then transferred to a 0.22 µM nitrocellulose membrane using a BioRad blotting system at a constant 50 V for 75 min. Ponceau stain was carried out to ensure effective transfer. The membranes were subsequently blocked for 1 h at room temperature in 1X TBS containing 5% (w/v) bovine serum albumin. Proteins were detected by incubation with primary antibodies; CD63 (abcam, ab271286, 1/1000), Calnexin (abcam, ab112995, 1/2000), Alix (abcam ab186728, 1/1000), HSP70 (abcam, ab181606, 1/1000), APOA1 (abcam, ab211472, 1/100) in blocking solution overnight at 4 °C. Due to the lack of availability of feline specific antibodies, the same panel was used for both human and feline EVs. They were then incubated for 4 h at room temperature. Following 3 X 5-min washes in TBS-T with 0.1% Tween, membranes were incubated in the appropriate dilution of IRDye800-conjugated goat anti rabbit IgG and IRDye680- conjugated goat anti-mouse IgG secondary antibodies (Li-COR Biosciences) diluted in 5% milk for 1 h at room temperature. The blots were then washed 5 times for 5 min. Proteins were visualised by scanning the membrane with an Odyssey Infrared Imaging System (Li-COR Biosciences) with both 700 and 800 nm channels.

### Mass spectrometry

Plasma EVs from fractions 7–10 were pooled and concentrated in duplicate. Concentrated EVs were resuspended in 20 µl PBS. A 40 µl aliquot of 8 M Urea/50 Mm Tris HCL was added to 20 µl of sample. The protein samples were reduced by adding 8 mM DTT, and mixing (thermomixer 1000 rpm, 30 °C) for 60 min and carboxylated by adding 20 mM iodoacetamide and mixing (thermomixer 1000 rpm, 30 °C) for 30 min in light exclusion. The samples were then diluted with 50 mM Tris HCL to bring the urea concentration below 2 M. It is important that the Urea concentration must be below 2 M to prevent inhibition of trypsin. Each sample was digested overnight with sequencing grade trypsin in a 1:20 enzyme to substrate ratio (Promega, V5111). Digestion was terminated by adding formic acid to 1% final concentration.

Following trypsin digestion, the samples were cleaned using C18 HyperSep SpinTips (Thermo Scientific). Samples were run on a Bruker timsTof Pro mass spectrometer (Bruker Daltonics, Bremen) connected to a Bruker nanoElute nano-lc chromatography system (Bruker Daltonics). Tryptic peptides were resuspended in 0.1% formic acid. Each sample was directly injected onto an Aurora UHPLC column (25 cm × 75 μm ID, C18, 1.6 μm, (Ionopticks) and was separated with an increasing acetonitrile gradient over 40 min at a constant temperature of 40 °C. In detail, after equilibration buffer b was increased from 2 to 30% between 0 and 33 min at a flow rate of 250 µl/min, from 33 to 34 min buffer b was increased from 30 to 95% and from 34 to 40 min buffer b was maintained at 95% to wash the column. The mobile phases used were buffer a (lcms grade water/0.1% formic acid) and buffer b (lcms grade acetonitrile/0.1% formic acid).

The mass spectrometer was operated in positive ion mode with a capillary voltage of 1600 V, dry gas flow of 3 l/min and a dry temperature of 180 °C. All data was acquired with the instrument operating in trapped ion mobility spectrometry (TIMS) mode. Trapped ions were selected for ms/ms using parallel accumulation serial fragmentation (PASEF). A scan range of (100–1700 m/z) was performed at a rate of 5 PASEF MS/MS frames to 1 MS scan with a cycle time of 1.03 s^20^.

### Proteomic data analysis and STRING analysis

The raw data was searched against the *Homo sapiens* subset for human samples and *felis catus* subset for feline samples of the Uniprot Swissprot database (reviewed) using the search engine Maxquant (release 2.0.2.0) using specific parameters for trapped ion mobility spectra data dependent acquisition (TIMS DDA). Each peptide used for protein identification met specific Maxquant parameters. Specifically, only peptide scores that corresponded to a false discovery rate (FDR) of 0.01 were accepted from the Maxquant database search. The normalised protein intensity of each identified protein was used for label free quantitation (LFQ).

Mass spectrometry data was analysed for similar proteins identified in both human and feline EVs using Microsoft Excel (Supplementary Information, Sect. 3). Subsequently, the top 100 proteins identified^21,22^ in the EVs were also compared to the human and feline protein identifications. To assess the function of these proteins and their networks, the gene names were entered into STRING^23^. In addition, the biological processes (Gene Otology) and functional networks associated with the proteins identified in EVs were recorded. Common KEGG pathways^24,25^ were identified to assign functional meanings to the proteomic identification.

### Field flow fractionation

Asymmetrical-flow field flow fractionation (AF4), using a Postnova AF2000 system (Postnova Analytics Ltd, Malvern, UK) was used for the analytical separation and size distribution determination of the EVs. The system was configured with UV (Postnova PN3211), refractive index (RI, Postnova PN3150) and multi-angle light scattering (MALS Postnova PN3621) detectors to obtain the concentration and size distributions of the eluting EVs. The separation was performed using the standard analytical channel equipped with a 350 µm spacer and a regenerated cellulose membrane (NovaRC, 10 kDa). The eluent was PBS pH 7.4, at a channel flow rate of 0.5 mL/min. The elution method detail is given in the supplementary information (Supplementary Information, Sect. 4). The samples were injected without dilution and the injection volume was 75 µL. The run time was 65 min, and the data was captured using NovaFFF software (vers. 2.2.0.1). Calculations of the size distributions were performed with both NovaFFF and NovaAnalysis (vers. 2103) software packages.

### Metabolite extraction

Plasma EVs from fractions 7–10 were pooled and concentrated in duplicate. EV samples were dried under a stream of nitrogen and resuspended in 25 µl ice-cold Ethanol/PBS (85:15, v/v). The resuspended samples were sonicated in an ice bath for 3 min and then snap-frozen in liquid nitrogen for 30 s. This was repeated for 2 cycles followed by centrifugation at 24,000×*g* for 5 min at 2 °C. The supernatant was collected for metabolomic analysis.

### AbsoluteIDQ® p180 assay and sample preparation

Metabolomic analysis was performed using a targeted metabolomics platform with the AbsoluteID® p180 assay (Biocrates Life Sciences, Innsbruck, Austria). Samples were prepared according to the manufacturers’ manual. The detailed preparation steps and measurement methods is described elsewhere^26,27^. Briefly, 10 µl of supernatant was added to the 96-well kit plate and the plate was subsequently dried under a stream of nitrogen for 30 min at room temperature. Then 50 µl of 5% phenyl isothiocyanate derivatization solution was added, incubated for 25 min at room temperature and dried for 60 min. Metabolites were extracted with 300 µl 5 mM ammonium acetate in methanol by shaking the plate for 30 min, and the 96 well plate was centrifugated at 500×*g* for 2 min. The eluate was diluted according to the manufacturers’ manual for LC–MS/MS run and flow injection analysis–tandem mass spectrometry (FIA-MS/MS) run.

### Sample analysis by LC–MS

The targeted metabolomics platform included a SCIEX QTRAP 6500plus mass spectrometer coupled to SCIEX ExionLC™ Series UHPLC capability. Metabolites were separated by a UHPLC column using 95% acetonitrile with 0.2% formic acid (mobile phase B) and 100% water with 0.2% formic acid (mobile phase A). LC–MS/MS analysis quantified 21 amino acids and 21 biogenic amines in positive mode. For the FIA-MS/MS, methanol was used as the running solvent and 40 acylcarnitines, 14 lysophosphatidylcholines (LPCs), 76 phosphatidylcholines (PCs), 15 sphingomyelins (SMs), and hexose (H1) were measured.

### Data processing

Amino acids and biogenic amines were quantified based on isotopically labelled internal standards and 7-point calibration curves in Analyst version 1.7.2 software. Other metabolites such as acylcarnitines, LPCs, PCs, SMs and hexose were measured semi-quantitatively by using 14 internal standards within the MetIDQ™ software (Biocrates). Metabolite concentrations are reported in µM.

## Results

### Nanoparticle tracking analysis (NTA) shows that small EVs isolated from human and feline plasma by SEC are enriched in fractions 7–10 with protein contamination also limited in these fractions

**Figure 2:**
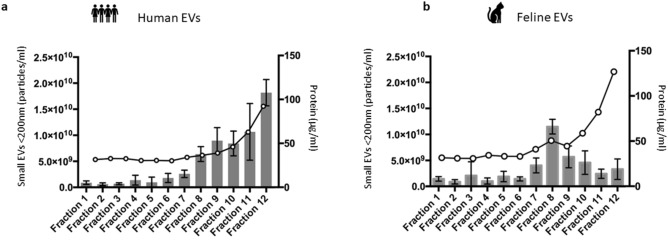
Nanoparticle tracking analysis (NTA) of the number of small EVs (< 200 nm) in each fraction isolated from the plasma of (**a**) humans and (**b**) felines by SEC. Protein concentration (-0-) of each fraction isolated by SEC from the plasma of (**a**) humans and (**b**) felines by BCA. **(a)** The enrichment of small EVs in human plasma within fractions 7–10 is shown. The protein concentration also increases in later fractions, with fractions 11 and 12 containing substantially more protein than previous fractions. **(b)** The enrichment of small EVs in feline plasma within fractions 7–10 is shown. The protein concentration increases in later fractions, with fractions 11 and 12 containing substantially more protein than previous fractions. This figure confirms that the majority of small EVs are contained in fractions 7–10, while fractions 11 and 12 from both human and feline plasma contain substantially more co-isolated contaminating protein than previous fractions. A high protein to particle ratio has been shown to indicate contamination by plasma proteins^5^.

Nanoparticle tracking analysis (NTA) and subsequent protein quantification allowed the identification of fractions 7–10, as key fractions for subsequent analysis of small EVs from both human (Fig. 2a) and feline plasma (Fig. 2b). NTA was carried out in duplicate on each fraction (1–12 inclusive) following EV isolation by IZON size exclusion chromatography. The mean number (particles/ml) of small EVs with a size of less than 200 nm is indicated by the bars and left y-axis. Error bars represent the SEM. The protein concentration was carried out in duplicate on each fraction (1–12 inclusive) following EV isolation by IZON size exclusion chromatography (SEC). Pierce BCA assay shows the mean total protein concentration in each fraction (µg/ml) indicated by the dots and the right y-axis. Figure 2a shows that small EVs from human plasma were enriched in fractions 7–10. Additionally, a sharp increase in the protein content of fractions 11 and 12 are evident. Similarly, Fig. 2b shows that small EVs from feline plasma were also enriched in fractions 7–10, with later fractions 11 and 12 also showing a sharp increase in protein content. Increased protein in the later fractions indicates co-isolation of contaminating plasma protein by SEC (iZOn Science). Nanoparticle tracking analysis and subsequent protein quantification (-0-) highlights fractions 7–10 as key fractions for subsequent downstream analysis of small EVs from both human and feline plasma.

### Size profile of EVs from human and feline plasma demonstrates isolation of the small EV subtype

**Figure 3:**
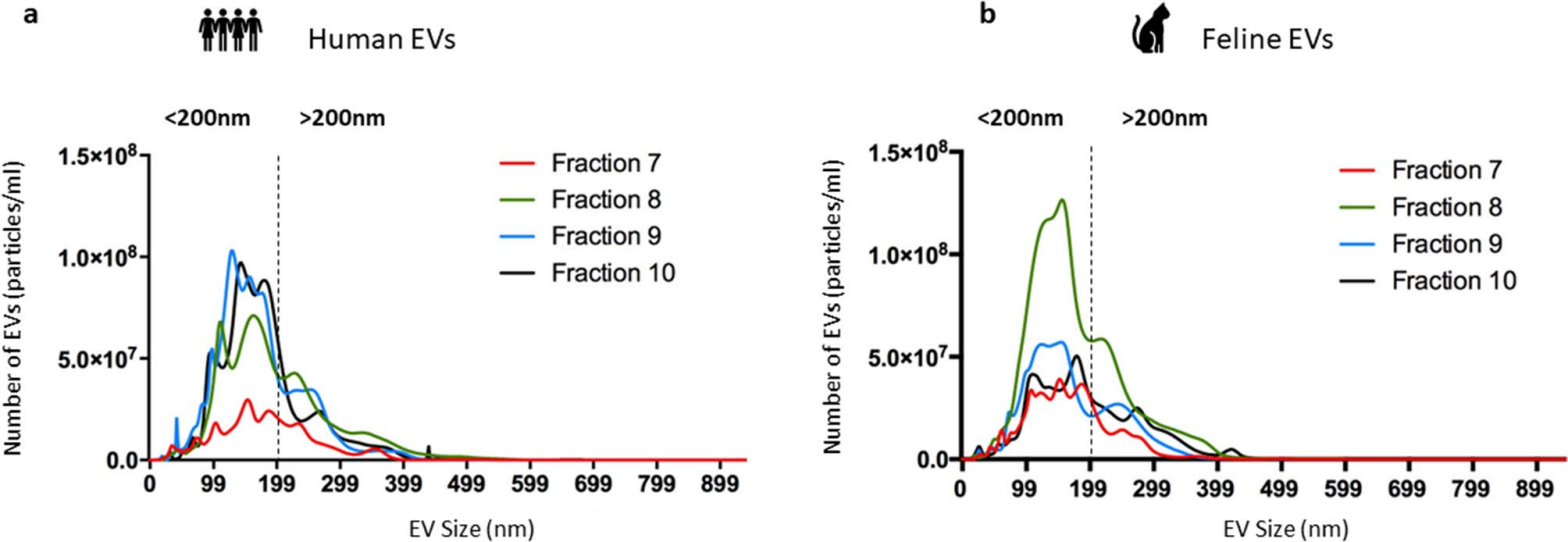
Nanoparticle tracking analysis of fractions 7–10 showing the size range and concentration of all EVs isolated from human and feline plasma. **(a)** NTA of particles isolated from human plasma shows that the peak in EV concentration is below 200 nm for fraction 7 (−) (mode: 168.0 ± 23.5 nm), fraction 8 (−) (mode: 170.0 ± 19.5 nm), fraction 9 (−) (mode 143.0 ± 15.8 nm) and fraction 10 (−) (mode: 176.3 ± 6.4 nm). A population of vesicles greater than 200 nm are also present in fraction 7 (D90: 286.6 ± 23.9 nm), fraction 8 (−) (D90: 347.6 ± 15.1 nm), fraction 9 (−) (D90: 275.0 ± 14.9 nm), fraction 10 (−) (D90: 300.9 ± 24.8 nm). (**b)** NTA of particles isolated from feline plasma shows the EV concentration is below 200 nm for fraction 7 (−) (mode: 110.0 ± 28.3 nm), fraction 8 (−) (mode: 172.0 ± 11.5 nm), fraction 9 (−) (mode: 137.0 ± 11.5 nm) and fraction 10 (−) (mode: 137.0 ± 20.4). A population of vesicles greater than 200 nm are also present in fraction 7 (−) (D90: 250.6 ± 8.7 nm), fraction 8 (−) (279.0 ± 17.4 nm), fraction 9 (−) (D90: 298.8 ± 13.0 nm), fraction 10 (−) (D90: 246.1 ± 28.7 nm).

Nanoparticle tracking analysis (NTA) showing the mean number of particles and size range of EVs isolated from the plasma of human (Fig. 3a) and felines (Fig. 3b) in fraction 7 (−), fraction 8 (−), fraction 9 (−) and fraction 10 (−). Figure 3 confirms that for both human and feline derived EVs, fractions 7–10 are enriched for the small EV subtype (less than 200 nm), but these fractions also contain a small number of vesicles greater than 200 nm.

### Transmission electron microscopy (TEM) visualisation of small EVs isolated from feline and human plasma

**Figure 4:**
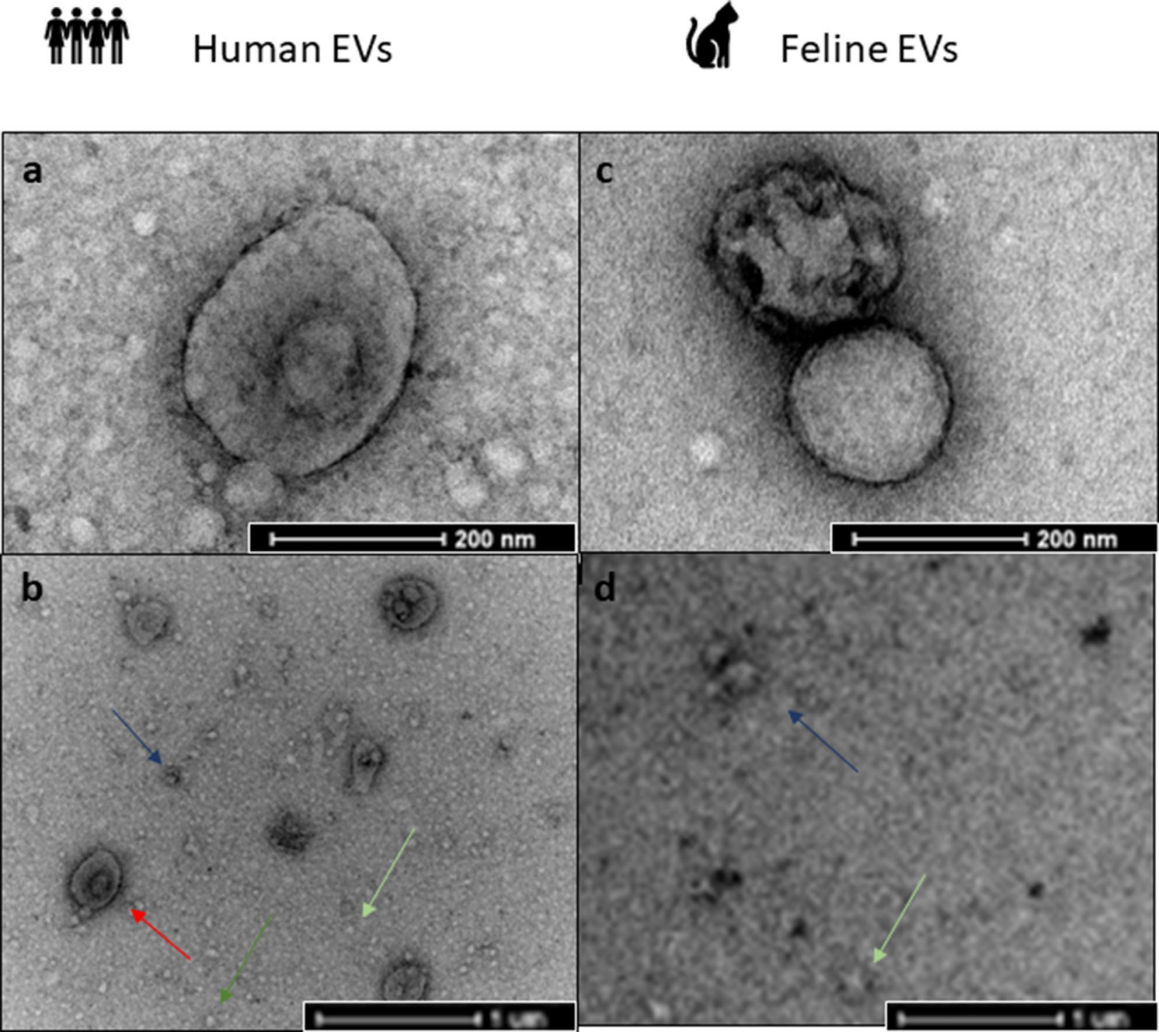
Transmission electron microscopy (TEM) showing the structure of human and feline EVs. **(a)** Human plasma derived EVs, (magnification × 87,000, scale bar 200 nm) show a small EV around 200 nm in size. **(b)** Human plasma derived EVs, (magnification × 16,500, scale bar 1 µm) show small EVs indicated in blue as well as a population of larger vesicles indicated in red. Lipoproteins are also visible in green. **(c)** Feline plasma derived EVs, (Magnification: × 87,000, scale bar 200 nm) shows an example of a small EV around 150 nm in size (blue). **(d)** Feline plasma derived EVs, (magnification × 16,500, scale bar 1 µm) show a lower density of small EVs (blue) confirming that there is observationally less EVs in feline plasma than human plasma. No larger EVs were visible, while lipoproteins are also visible in green.

Using electron microscopy, cup-shaped vesicles were visualised with a morphology and size compatible with EVs (Fig. 4a,c). The size distribution of particles seen in Fig. 4 show that human EV populations contain two distinct subpopulations. A small EV population (blue arrow) is evident as well as a population of larger vesicles (red arrow). Feline EVs imaged in Fig. 4d contain a population of small EVs only. Figure 4 also demonstrates co-isolation of lipoproteins (green arrow) from both human and feline plasma.

### Confirmation of EV enrichment by recognised EV markers

**Figure 5:**
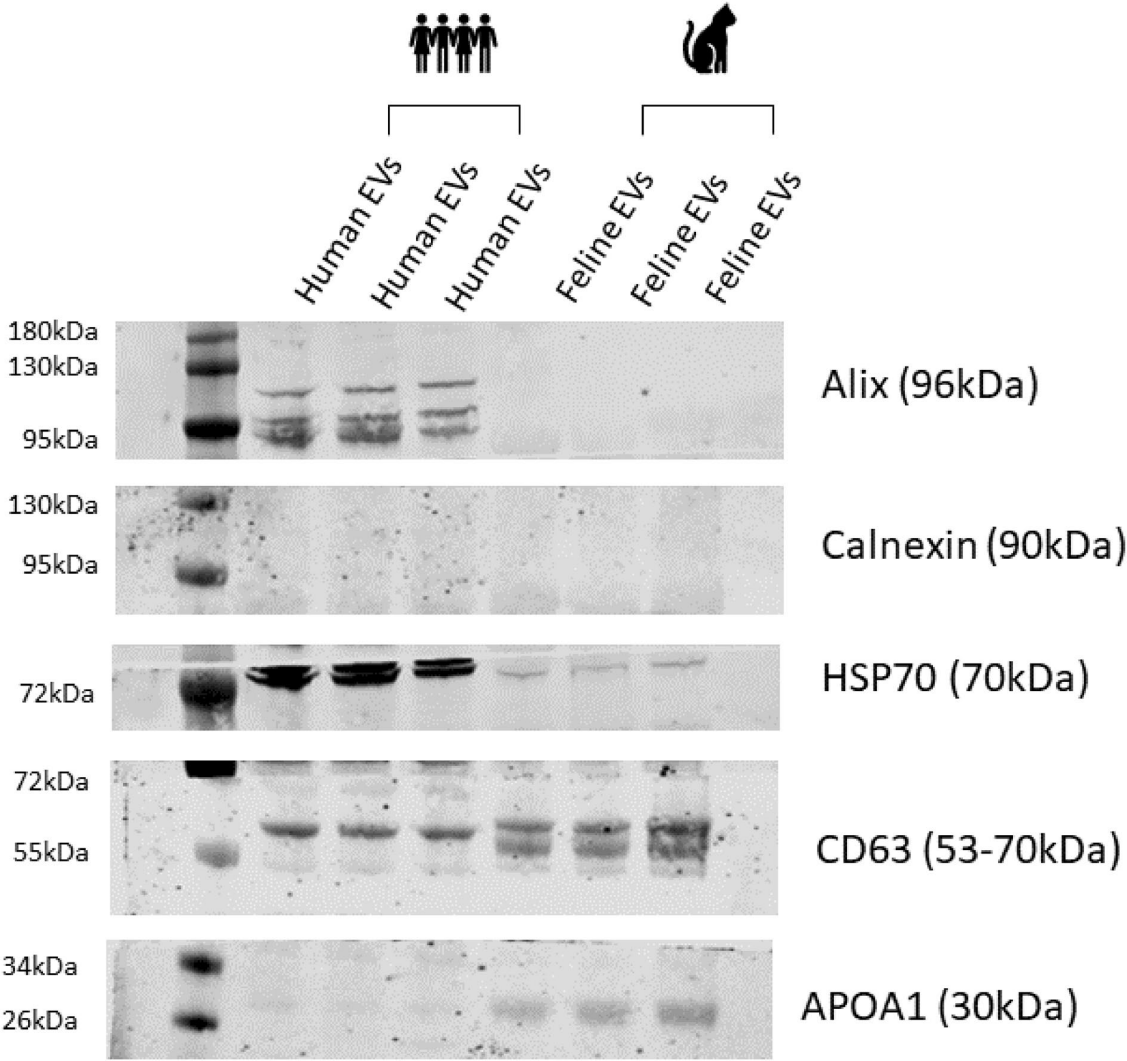
Western blot analysis confirming the recognised EV markers according to the MISEV guidelines 2018^5^. Western blot analysis shows the protein content of EVs isolated from human plasma (n = 3 replicates) and feline plasma (n = 3 replicates). Molecular weight markers are also shown. EVs from both humans and felines concentrated, lysed, and subjected to western blot for recognised EV markers; Alix, Calnexin, HSP70, CD63 and APOA1. Representative images are shown.

Western blot analysis of pooled and concentrated EV fractions 7–10 from human and feline plasma were analysed in triplicate. Table 3 in the MISEV guidelines 2018^5^ highlights three categories of markers which must be analysed in all bulk EV preparations to confirm the presence of EVs (Category 1 and 2) and ‘*negative controls’* relevant to particular subtypes of EVs are also suggested. Category 1 detects transmembrane or GPI-anchored proteins, demonstrating the EV specific lipid-bilayer structure. Category 2 displays the presence of cytosolic proteins or periplasmic proteins showing that the preparation of lipid bilayer structures encloses intracellular material, as expected for any EV. Category 3 represents major constituents of non-EV structures often co-isolated with EVs. Evaluation of their purity helps to assess the degree of purity in the EV preparation. Western blot analyses of known EV markers are shown in Fig. 5. From category 1, CD63 is present in EVs from both human and feline plasma. From category 2, HSP70 is present in both human and feline plasma, however Alix is present in human plasma only. From category 3, APOA1 is present in both human and feline EVs, detailing that lipoprotein is being co-isolated with EV particles. Category 4 is evaluated to be able to claim that the population is mainly composed of small EVs. Endoplasmic reticulum proteins are not enriched in the small EV populations, therefore *the absence of* the endoplasmic reticulum protein, calnexin is indicative of an enriched small EV population^5^.

### Mass spectrometry demonstrates similar protein profile of human and feline EVs

**Figure 6:**
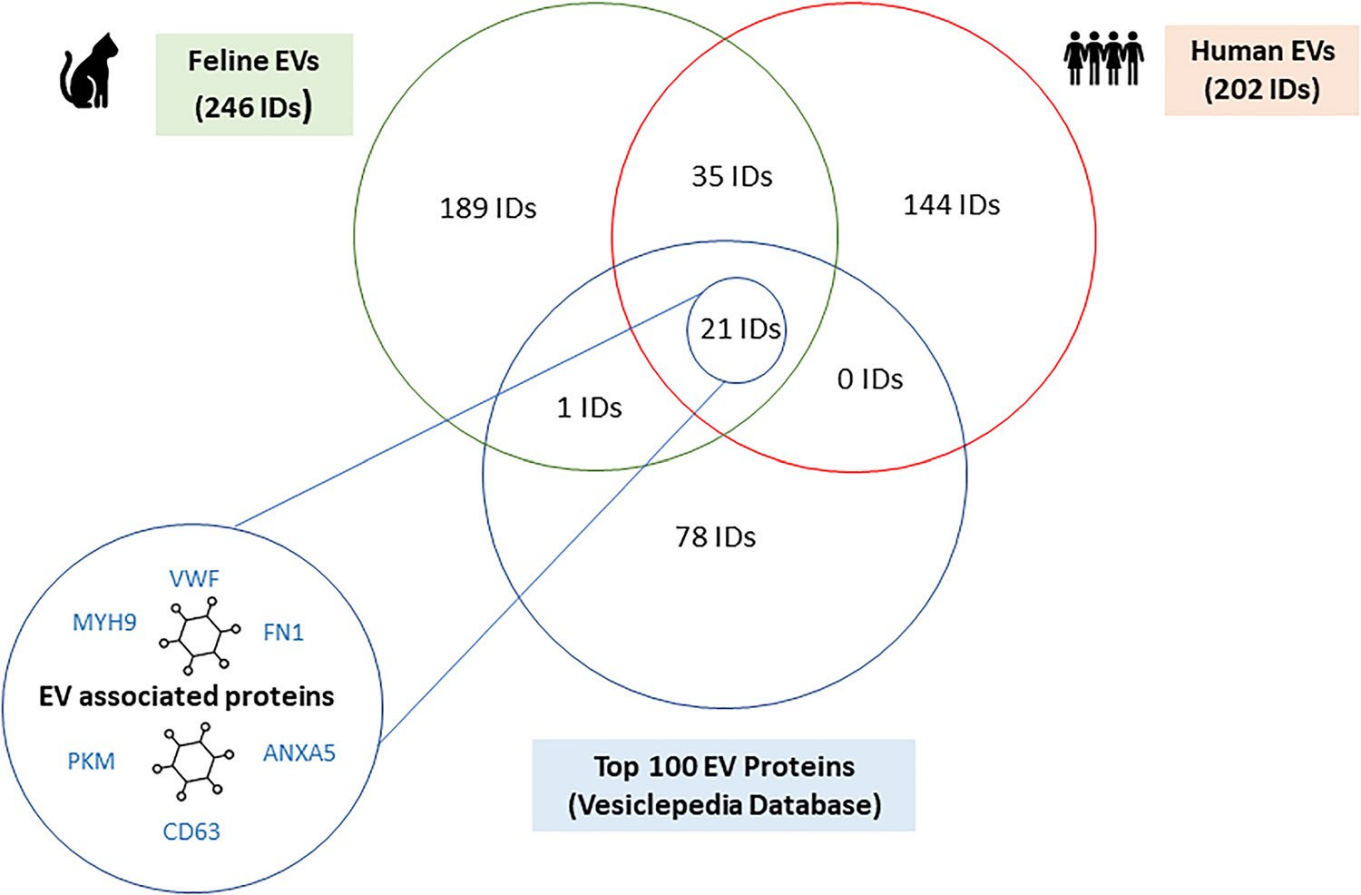
Venn Diagram showing the number of proteins identified by mass spectrometry (LC–MS/MS) in EVs isolated from human and feline plasma. Common proteins identified in both human and feline EVs included 21 proteins from the top 100 proteins present in extracellular vesicles (EVs) as identified by the Vesicplepedia database^21,22^. Additionally, 35 IDs were present in both human and feline EVs isolated by SEC. Some examples of EV associated proteins include VFW, CD63, ANXA5, FN1 and PKM (Supplementary Information).

EVs from fractions 7–10, were pooled and concentrated in triplicate. Following digestion, their contents were analysed by LC/MS–MS. Peptides were identified using a Bruker timsTof pro mass spectrometer. LC/MS–MS showed 86 common proteins (IDs) in human and feline EVs (Supplementary Information, Sect. 3). The protein identifications from human and feline EVs were also compared with the Vesiclepedia database. Figure 6 shows that common proteins identified in both human and feline EVs, included 21 proteins from the top 100 proteins present in extracellular vesicles (EVs) as identified by the Vesicplepedia database^21,22^. STRING analysis of protein IDs were carried out to establish the similarities and differences in human and feline EV protein function. Statistical and enrichment data demonstrates that proteins identified in both human and feline EVs have similar functions. Feline protein IDs (246 IDs) were searched against the *felis catus* database using STRING. The top 5 biological processes (gene otology) and KEGG pathways associated with feline plasma derived EVs are reported (Table 1). Statistical and enrichment data was also analysed in Table 1. Human protein IDs (202 IDs) were searched against the *homo sapiens* database in STRING. The top 5 biological processes (gene otology) and KEGG pathways associated with human plasma derived EVs are reported. Statistical and enrichment data was also analysed.

**Table 1:** Functional classification of proteins identified by LC–MS/MS.

246 proteins identified in feline extracellular vesicles by LC–MS/MS			
Gene Otology (Biological Processes)	Count in network	Strength: (Log10 observed/expected)	False discovery rate (p-value)
→ Positive regulation of protein processing in phagocytic vesicle	2 of 2	2.34	0.0304
Blood coagulation, fibrin clot formation	3 of 4	2.21	0.0011
Proteasomal ubiquitin-independent protein catabolic process	6 of 25	1.72	0.0000056
Acute phase response	5 of 27	1.6	0.00015
Complement activation, classical pathway	9 of 54	1.56	0.000000129

KEGG Pathways	Count in Network	Strength: (Log10 observed/expected)	False discovery rate (p-value)
→ Complement and coagulation cascades	16 of 73	1.68	4 × 10^–19^
Proteasome	5 of 40	1.43	0.00011
→ Staphylococcus aureus infection	7 of 66	1.36	0.0000482
→ Systemic lupus erythematosus	8 of 78	1.35	0.0000000953
African trypanosmiasis	3 of 29	1.35	0.0156

202 proteins identified in human extracellular vesicles by LC–MS/MS			
Gene otology (biological processes)	Count in network	Strength: (Log10 observed/expected)	False Discovery Rate (p-value)
Negative regulation of very low-density lipoprotein particle remodelling	3 of 3	2.13	0.00050
→ Positive regulation of protein processing in phagocytic vesicle	2 of 2	2.13	0.0113
Positive regulation of neurofibrillary tangle assembly	2 of 2	2.13	0.0113
Negative regulation of cholesterol import	2 of 2	2.13	0.0113
Peptidyl-cysteine s-trans-nitrosylation	2 of 2	2.13	0.0113

KEGG pathways	Count in Network	Strength: (Log10 observed/expected)	False discovery rate (p-value)
→ Complement and coagulation cascades	28 of 82	1.58	7.43 × 10^–24^
→ Staphylococcus aureus infection	17 of 86	1.35	5.24 × 10^–15^
Pertussis	14 of 74	1.33	7.03 × 10^–12^
Cholesterol metabolism	8 of 48	1.27	0.000000338
→ Systemic lupus erythematosus	9 of 93	1.12	2.49 × 10^–6^

When the top 5 biological processes (Gene Otology) and KEGG pathways associated with human and feline protein IDs were compared, both humans and felines showed an enrichment in proteins associated with one similar biological process and three common KEGG pathways (indicated by →)^24,25^. *Count in Network* indicates the number of proteins in the network associated with a particular term. *Strength* measures how large the enrichment effect is (Observed = the number of proteins present in the network that are annotated with a specific term, expected = the number of proteins we expect to be annotated with this term in a random network of the same size). *False Discovery Rate* measures the significance of the enrichment. P-values shown have been corrected for multiple testing within each category using the Benjamini–Hochberg procedure.

### Asymmetrical flow field flow fractionation (AF4) confirms size differences in subpopulations of EVs from human and feline plasma

**Figure 7:**
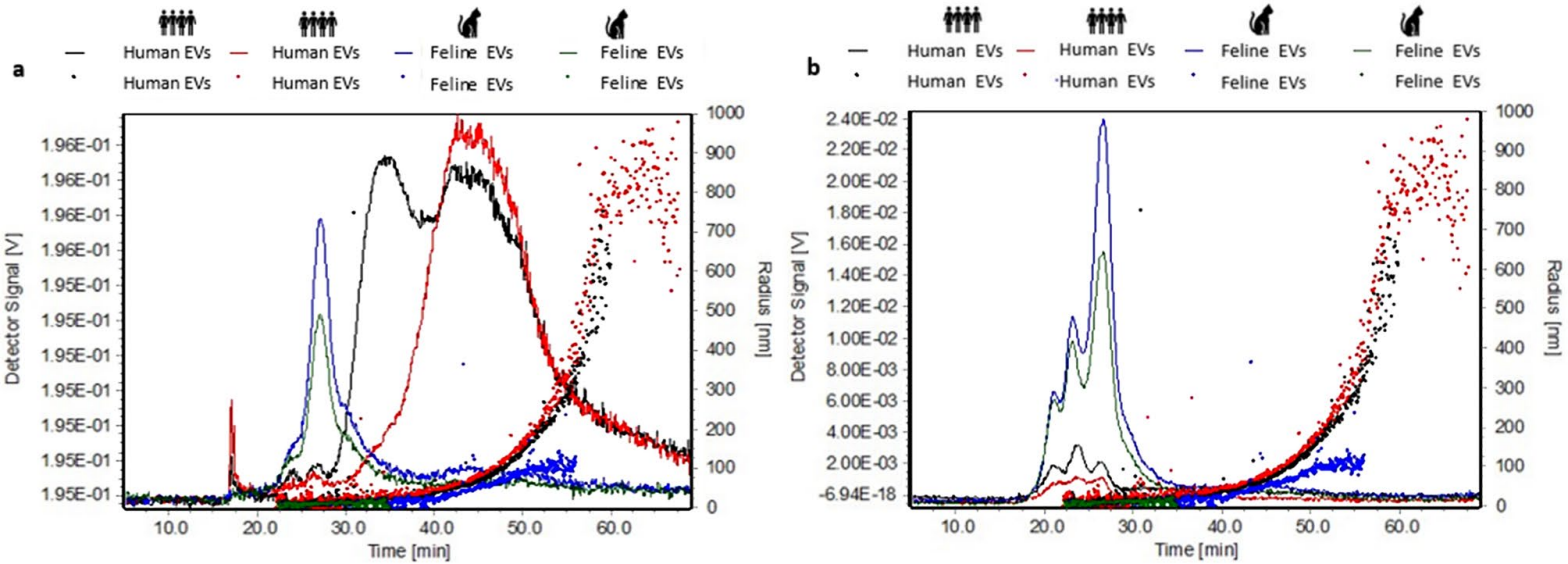
Asymmetrical flow field flow fractionation (AF4) of human and feline EVs. Asymmetrical field flow fractionation (AF4) was carried out on human and feline EVs. **(a)** Shows 90° light scatter signal (full lines, left hand scale) overlayed on the radius of gyration (dotted lines, right hand scale). Red and black lines represent the human EVs showing two distinct populations of EVs. One in the small EV range (< 200 nm) and another population of larger particles (> 200 nm). In contrast, the feline EVs indicated by blue and green lines show peaks that indicate only a small EV population only (< 200 nm). Radius of gyration (Rg) determined from light scattering signals was recorded from human and feline EV samples. These size differences show that the density of human EVs is different to the density of feline EVs, suggesting that their structure and shape are different. **(b)** Shows the radius of gyration (dotted lines, right hand scale) overlaid on UV detector signal (full lines, left hand scale) which shows the early eluting protein populations of the samples. The protein pattern of the human EVs (shown in black and red) was similar to feline EVs (blue and green).

Asymmetrical flow field flow fractionation (AF4) was carried out on human and feline EVs isolated from plasma by SEC. In Fig. 7a, 90° light scatter is overlaid on the radius of gyration (right y-axis) as detected by multi angle light scattering (MALS). Elution time is on the x-axis. Human EVs show two distinct peaks demonstrating that there are two populations of EVs scattering light in the human EV samples. The dotted lines represent the radius of gyration as detected by MALS. Light scatter indicated by the lined graph, shows one population of feline EVs strongly scattering particles with the low radius of gyration indicated by the dotted lines. The radius of gyration (Rg) data shows that the density and/or shape of human EVs is different to the corresponding feline EVs that elute at the same volume. In Fig. 7b the UV detector signal measuring the amount of UV light absorbed by the samples, is overlaid on radius of gyration (Rg). The UV signal is absorbed by protein. Early eluting proteins are detected in both human and feline samples corresponding with small EV populations. The shape of the UV elution profiles is similar for both humans and felines, suggesting similar protein composition.

### Plasma derived EVs are metabolically similar from both humans and felines

EVs isolated by SEC were subjected to metabolomic profiling. Metabolomic analysis (Table 2) identified 93 metabolites in human EVs, and 74 metabolites in feline EVs. This suggests that metabolically, plasma derived EVs are similar from both species. Amino acids were not detectable in the current samples analysed in either type of EV.

**Table 2:** Identification of metabolites in human and feline EVs.

Metabolites	Human EVs	Feline EVs
Amino acids	X	X
**Biogenic amines**		
Spermidine	✓	✓
Spermine	✓	✓
Putrescine	✓	X
Taurine	✓	X
LPCs	✓	✓
PCs	✓	✓
SMs	✓	✓
Hexose	✓	✓

Amino acids were not detectable in the current samples analysed. Four biogenic amines, spermidine, spermine, putrescine, and taurine were identified in human EVs, and spermidine and spermine were also identified in feline EVs. Many lipids such as LPCs, PCs, and SMs and the sum of hexose were identified in both human and feline EVs. The detailed metabolites identified in human and feline EVs are listed in the supplementary information (Supplementary Information: Sect. 5).

X < limit of detection. ✓ > limit of detection.

*LPCs* lysophosphatidylcholines, *PCs* phosphatidylcholines, *SMs* sphingomyelins.

## Discussion

Extracellular vesicles (EVs) are found in the bloodstream during normal and pathological conditions. EVs carry cargoes such as lipids, proteins, and RNA, which represent their parent cell. Studies of EVs have recently emerged as a potential mechanism of studying diagnostic tools, therapeutic targets, and delivery systems. The interest in EVs has been highlighted by the International Society for Extracellular Vesicles (ISEV) and the focus on research acknowledged by position papers detailing the minimal information required for EV isolation and characterisation^2,5^. However, despite these recommendations, research is continuously evolving in the field. Most recently, Mathieu et al., demonstrated that molecular and function discrimination of exosomes is possible in any cell type by monitoring CD63 and CD9 trafficking^4^. In addition, Crescitelli et al., have published a protocol to detail the isolation and characterisation of extracellular vesicle subpopulations from tissues^28^. The published EV studies are extremely diverse and span a wide variety of biological areas from allergenic inflammation^29^ to osteoarthritis^30^. However, most EV studies focus on human diseases only^5^.

Comparative medicine is an aspect of “*one health*” which relates the biological similarities and differences among species to examine naturally occurring diseases in humans and animals to better understand the mechanism of disease for both groups. Although the research “*niche*” is not focused on one particular disease or discipline, the shift to a “*one health*” mindset holds significant clinical potential for patients, humans and animals alike^31^. Comparative medicine also often allows basic scientific research to be translated to the clinical settings by the discovery of a common thread to decipher pathophysiological process between humans and animals. To date, EV studies involving veterinary patients have been somewhat limited. For example, expression of microRNAs contained in the EVs from dogs, have resulted in the identification of biomarkers capable of differentiating glioma from other intracranial diseases^32^. Moreover, Pollott et al., have published a comparison of different methodologies for the measurement of EVs from cow’s milk^33^. Interestingly, there have not been any published studies examining the role of EVs in feline pathophysiology.

The biological similarity between humans and felines^34^ has been discussed at length. A paper by Baral et al., investigated whether human quality control materials may be replaced with feline plasma for common biochemical analytes. They showed that human quality control material is commutable to feline plasma pools within the ranges tested^35^.

However, the similarities and differences in EVs from these groups have not been investigated. Additionally, the ISEV are yet to detail the applicability of their MISEV guidelines to veterinary studies, particularly studies involving small animals^5^. Before feline derived EVs isolated from liquid biopsies may be studied in the pathological setting, we must first establish the normal or non-diseased characteristics of feline plasma derived EVs. Therefore, the aim of this study was firstly to investigate whether the guidelines laid out by the ISEV for the isolation and characterisation of EVs^5^ are in fact applicable to small animals such as felines, and secondly to establish the similarities and differences in EV structure and cargoes between human and feline veterinary cohorts.

In the current study, a quantitative and qualitative comparison of EV populations isolated from plasma of healthy humans and felines was undertaken (Fig. 1). Unsurprisingly, EVs from felines may be isolated and characterised according to the MISEV 2018 guidelines^5^. However, some adjustments may still be advocated for. Interestingly, the EVs from humans and felines were remarkably similar in size, shape, protein content and metabolomic content. Plasma was collected from 3 healthy humans and pooled, it was then divided into 500 µl aliquots and frozen. Plasma from 20 felines was collected, pooled, and subsequently divided into 500 µl aliquots and frozen. Plasma from healthy individuals was pooled to eliminate any biological differences between replicates. Our aim was to compare a ‘general’ human EV signature to a ‘general’ feline EV signature. We are cognisant of the potential that EVs have for mediating interactions and its potential effect on pooled samples. However, pooling samples seemed logical to reduce individual variation for between species comparison of EVs and increases the volume of plasma available for analysis. All plasma from humans and felines was collected as part of routine clinical testing or blood donation procedures and would have been discarded otherwise. Pooled plasma from routine diagnostic testing was used to (i) reduce any unpleasant procedures for humans and felines and (ii) to show that EV studies may be easily translated to the clinic.

Table 1 of the MISEV guidelines^5^ details the considerations for EV separation and enrichment. As there has been no single, optimal method identified for the isolation of EVs from human plasma based on downstream applications or scientific questions, we chose a low recovery, high specificity method to isolate EVs. IZON SEC was used to isolate EVs from 500 µl aliquots of human and feline plasma. Our first objective was to investigate whether small EVs isolated by SEC from felines are contained in the same fractions as small EVs isolated by SEC from humans. Fractions were collected immediately following addition of plasma, no void volume was collected. Nanoparticle tracking analysis (NTA) was subsequently carried out on each fraction in duplicate. Five technical replicates were recorded for each. Figure 2 shows the mean number of small EVs (< 200 nm) in each of fractions 1–12 isolated by SEC. In the case of both humans and felines, the majority of small EVs are eluted from fraction 7 onwards. In addition, the total protein concentration was examined according to Table 2 in the MISEV 2018 guidelines^5^ for each fraction in duplicate. Figure 2 shows that there is a sharp increase in the amount of protein in fractions 11 and 12. As there is no perfect protein quantification method, the total protein amount and particle number may be used as a measure of purity and reliability of the quantity. A high particle to protein ratio has been shown to indicate contamination by plasma proteins. Therefore, based on this analysis, it was concluded that fractions 7–10 isolated from human and feline plasma are key fractions for the analysis of small EVs (< 200 nm) and indicates that their contamination is limited.

Figure 3 focuses on the mean size and mean concentration of EVs contained in key fractions identified by Fig. 2. The size range and concentration of all particles isolated from human and feline plasma were analysed in duplicate by NTA, where five technical replicates were carried out per run. Fractions 7–10 isolated from human plasma show a sharp concentration peak less than 200 nm in size. The modal size of each fraction also confirms that most particles in these fractions are less than 200 nm in size. Similarly, fractions 7–10 isolated from feline plasma show similar sharp concentration peaks less than 200 nm in size. The modal size of each fraction also confirms that most particles in these fractions are less than 200 nm in size. Even though we have shown that fractions 7–10 are enriched for the small EV subtype, the graphs in Fig. 3 also show that there are some larger EVs present. The MISEV 2018 guidelines^5^ detail that it is virtually impossible to isolate a pure population of EVs. Therefore, it is expected that non-EV particles are co-isolated with small EVs. To analyse these larger particles, the mean D90 value was recorded for each fraction, detailing that 90% of particles detected by NTA were smaller than this D90 value. The D90 value was less than 300 nm for most of the fractions isolated from both human and feline plasma confirming that the fractions of interest contain most EV particles concentrated in the small EV size range. In conclusion, Fig. 3 confirms the enrichment of small EVs isolated from human and feline plasma by SEC and shows that there is no statistically significant difference in the number of EVs isolated from human plasma compared to feline plasma.

MISEV 2018 guidelines explains that single particle analysis by TEM provides information on the individual EVs present in bulk preparations^5^. TEM allows the visualisation of single EVs; however, TEM is difficult to exploit in a quantitative manner as there is often not enough particles present in very pure populations to reach statistical power. It is also important to consider that dehydration of samples for TEM also makes it more difficult to see the structure and composition of EVs. Therefore, taking these limitations into account, we used TEM for the purposes of this study, to assess whether EVs from human and feline plasma are similar in shape. Figure 4 shows that the structure of human and feline EVs are similar, indicating the spherical morphology associated with small EVs. While images **b.** and **d.** show a snapshot of the entire population of EVs. Larger particles are evident in **b**, that are not present in **d**. This does not correlate exactly with the results shown in Fig. 3, where both populations were shown to contain a subset of larger particles. Interestingly, the large particles may in fact be small EVs contained inside larger vesicles. A study of plasma derived EVs from other species such as dogs would be interesting to determine whether the difference in large vesicle population is specific to humans and felines. Alternatively, as TEM is carried out on a very small subset of the population, the larger particles may simply not have been added to the grid for imaging.

Figure 5 represents a western blot analysis, confirming the recognised EV markers according to the MISEV guidelines 2018^5^. While MISEV do not propose protein markers that will characterise specifically each EV subtype, the guidelines are said to reflect an evolving understanding of the subtypes of EVs and their associations with other entities^5^. There is also no universal loading or negative control relevant to specific EV subtypes which can make western blot of EVs challenging. Additionally, due to the low total protein concentration in each fraction, assessment of protein content is extremely difficult in relatively pure EV samples such as those shown in Fig. 5. Fractions 7–10 containing EVs from human and feline were pooled, concentrated, and lysed. Western blot analysis was carried out on human and feline EVs in triplicate. The transmembrane protein CD63 is present in EVs from human and feline plasma, cytosolic protein HSP70 is present in both human and feline EVs, however Alix is not present in feline EVs. As EV research on felines and indeed other veterinary patient cohorts is very much in its infancy, there are no specific antibodies available for western blot of feline proteins. This most likely accounts for a potential reason that Alix could not be detected in feline EVs. This also highlights the need for specific comparative medicine research as well as the need to diversify the MISEV guidelines^5^ to include EV characterisation for animals other than humans. APOA1 was measured to acknowledge some contamination by lipoproteins, as it is widely known that it is near impossible to obtain a pure population of EVs^5^. Lipoproteins are co-isolated with EVs and often outnumber them. Western blot analysis indicates the levels of APOA1 in human and feline EVs, suggesting that relatively similar lipoproteins are co-isolated with EVs following IZON size exclusion chromatography. To date, one published study is available detailing the properties of feline lipoproteins. Demacker et al., (1987) showed that feline serum lipoprotein composition may be quite different to that of humans. They demonstrated that felines contain five times as much high-density lipoproteins (HDL) as low-density lipoproteins (LDL), with a considerable number of beta-migrating particles also proteins. The LDL of cats and man however had a similar chemical composition, but feline LDL had a higher negative charge, were smaller and contained APOA1^36^. As LDL is most commonly co-isolated with EVs from human plasma, the reported similarities between the LDL profile in humans and felines suggests that comparing the EV profile of humans and felines would not be affected by differences in LDL properties in felines. Finally, Calnexin was evaluated to establish that the population is composed mainly of small EVs. The absence of this endoplasmic reticulum protein in both human and feline EVs has been shown to be indicative of a small EV population^5^.

Mass spectrometry has recently become economical and accessible, allowing a fingerprint-type assessment of many proteins in just one run. However mass spectrometry analysis of EVs has proven challenging. One of the main aspects influencing mass spectrometry analysis of EVs, is the method of EV isolation and concentration. This has been shown to have a major effect on subsequent proteomic analyses^37^. In Fig. 6, we have shown that the proteomic profile of human and feline EVs are remarkably similar with 55 common proteins identified (Supplementary information, Sect. 3). These were also shown by STRING analysis to be associated with similar KEGG pathways and subsequent biological processes (Table 1). This confirms the relevance of comparative EV studies in both human and feline diseases. Despite the lack of EV studies in felines, like humans, we have shown that EVs isolated from the plasma of felines hold biological relevance (Table 1). Additionally, functional enrichment analysis has confirmed that EVs from humans and felines display a similar protein profile. Further studies comparing the proteomic content of EVs isolated from the plasma of felines with pathologies to healthy felines may hold potential for biomarker discoveries in veterinary medicine. Even though we have confirmed that our small EV particle isolation is relatively pure and shown clear similarities between EVs isolated from human and feline plasma, it is interesting that 21 identical proteins were identified when humans and felines were compared to the top 100 EV proteins on Vesiclepedia. These included known EV markers such as CD63 and CD9^5^. The lack of EV studies in non-human mammals may be contributing to this relatively low number, as it was shown that 21 common proteins were identified when human EV proteins were compared to the top 100 EV proteins. This highlights the need for further comparative studies and sharing of results on EVs from other animals. Origin, isolation techniques and concentration methods are also potential reasons for the relatively low similarity between human and feline EVs and the top 100 proteins^21,22^. It is known that most EV studies to date have studied EVs from cell lines. These relatively homogenous populations would therefore be expected to contain different proteins than the heterogenous populations which are more commonly isolated from plasma. In addition, the method of EV isolation has also been shown to influence the proteomic content of EVs. Less pure methods such as ultracentrifugation as described by the MISEV guidelines^5^ typically contain more contamination by plasma proteins than EVs isolated by SEC^37^. Upon literature search, it has been shown that most EV studies have used an ultracentrifugation method to isolate EVs, therefore it can be deduced that the top 100 proteins^21,22^ recorded in EV studies to date may in fact be contaminants from less pure EV preparations. Therefore, the EV community would benefit from more specific proteomic lists and databases which detail the origin, isolation technique and concentration method of EVs prior to proteomic analysis to encourage rigorous and standardised EV research.

Field flow fractionation (FFF), is an upcoming technique of interest for the EV research community. Evidence has shown subpopulations of EVs work together to elicit biological responses^4^. Most studies to date have focused on one EV subtype^5^. In this study, our aim was to analyse small EVs (< 200 nm) isolated from both human and feline plasma and to establish a baseline EV signature for further comparison of EVs in the pathological setting. While NTA confirmed enrichment of small EVs, TEM demonstrated that there were a population of larger vesicles also being isolated by IZON SEC. To investigate the true nature of the population of EVs being studied, highly sensitive analysis by asymmetrical flow field flow fractionation (AF4) was carried out. While FFF can be used both as a physical isolation technique^38^ and as an analytical separation technique. In the later mode it is analogous to SEC in that it separates the analytes into a size ordered elution profile. The major differences are that it does not require a stationary packing phase or sample filtration prior to analysis. This means it can be applied to a wider range of biological solutions and/or suspensions containing molecules, vesicles and particles from the low nm range up to several microns in size. Pre-isolated EVs were analysed to ensure continuity and allow for comparison across other techniques. In Fig. 7a 90° light scatter is overlaid on the radius of gyration (right y-axis) as detected by MALS. Elution time is on the x-axis. Red and black lines represent the 90-degree light scatter of human EVs. Two distinct peaks are shown. One at around 18 min and another much larger peak at around 40 min, showing that there are two populations of EVs scattering light in the human EV samples. Dotted lines represent the radius of gyration as detected by MALS. At around 18 min, the radius of gyration is low showing that this scattering is due to a population of small particles, whereas for the large scattering peak seen at approx. 40 min, the radius of gyration remains relatively low suggesting an increase in the number of particles. Towards the tail of the graph, the radius of gyration increases, whereas the light scattering decreases, suggesting elution of a small number of increasingly large particles. There is some variability from sample to sample, but the essential features are unchanged. Feline samples are shown in blue and green. Light scatter indicated by the lined graph shows strongly scattering particles eluted at around 25 min with low radius of gyration indicated by the dotted lines, suggesting more such particles are present than in human samples. Based on this experiment, there is one distinct population of EVs present in feline samples in the small size range. There is apparently less variability from sample to sample than for human samples. Light scatter of feline samples is different to human samples, suggesting that the structure of human and feline EV populations differ. This may be due to differences in the composition of EV membranes, differential glycosylation, or differences in their cargos. The radius of gyration (Rg) data shows that at a given retention time the human EVs are larger. As the retention volume is proportional to hydrodynamic size, this indicates that for a given hydrodynamic size, the human EVs have a larger Rg. The density and/or shape of human EVs is different to the corresponding feline EVs that elute at the same volume. For example, the human EVs could be elongated compared to the feline EVs. Further studies would be required to determine more details of this difference.

In Fig. 7b UV detector signal measuring the amount of UV light absorbed by the samples, is overlaid on Radius of gyration. Human EV samples are in black and red, while feline EV samples are in blue and green. UV signal is absorbed by protein. UV detector signal with radius of gyration shows a population of small EVs with a UV peak between 19 and 35 min for human samples, where most small particles are eluted. These early eluting proteins are also detected in feline samples corresponding with small EV populations. However, there is more UV signal detected in feline samples. Figure 7a suggests that there are more EVs in human samples in this elution/size range, so it is difficult to comment on the relative protein content of these human and feline EVs. The shape of the UV elution profiles is similar for both humans and felines, suggesting similar protein composition. Biologically, these results are relevant as comparative medicine and ‘one-health’ approaches to EV research expands. When examining EV populations in veterinary cohorts and attributing characteristics to biological functions, it is important to note that direct comparison of EV signatures across animal species may not be directly transferrable. Further comparative analysis of EVs from other biological fluids may indicate whether the characteristics observed in Fig. 7 are specific to plasma. Additionally, further comparative studies may also indicate whether the differences observed between human and feline EVs may be attributed to the size differences or differential complexity of the species.

Metabolomic analysis shown in Table 2. Identified 93 metabolites in human EVs and 74 metabolites in feline EVs. This suggests that metabolically, plasma derived EVs are similar from both species. Unfortunately, amino acids were not detectable in the current samples analysed in either type of EV. A higher number of EVs may be necessary for the measurement of these metabolites, highlighting another limitation of EV studies from small volumes of plasma in the clinical setting. These are an important class of metabolites and future work is warranted in optimising the amount of EVs to measure these potential amino acids. Lipids are essential molecular components of EVs. However, our knowledge about the lipid composition and their function in EVs from human and feline plasma is limited^39^. Further comparison between the lipid profile of EVs from the plasma of humans and felines with pathologies, is warranted.

## Conclusion

In conclusion, we have isolated EVs from the plasma of humans and felines by iZON size exclusion chromatography (SEC) and confirmed the isolation using several commonly used methods. Further analysis was carried out by examining the proteomic and metabolomic content of EVs from both groups. To our knowledge, this is the first study to investigate whether EVs can be isolated from feline plasma and to investigate whether these EVs are similar to EVs from human plasma. Additionally, this study also provides a useful baseline for future studies of EVs from the plasma of felines with pathologies. Results showed that similar numbers of small EVs were isolated from both human and feline plasma. However, human EV samples contained larger EV particles co-isolated with small EV populations. The protein signature of EVs from both humans and felines was similar and KEGG analysis showed that EVs hold biological relevance in disease in both groups. Metabolic analysis showed similarities between EVs from human and feline plasma, but also highlighted the potential role of metabolites as biomarkers of disease. Isolation and characterisation of EVs from humans and felines was carried out according to the MISEV 2018 guidelines and further examination of the EV content was carried out by metabolomic and proteomic analysis, showing that EVs isolated from the plasma of both humans and felines are similar, but not identical. Therefore, this study serves as a useful baseline for comparative studies of EVs in veterinary medicine, with relevance for studying EVs in feline pathobiology.

## Supplementary Information

Supplementary Information.

## Data Availability

The datasets generated and/or analysed are available from the corresponding author upon request. Data are available via ProteomeXchange with identifier PXD034369.
