# Supplementary material for: A comparative analysis of extracellular vesicles (EVs) from human and feline plasma
Source: Sci Rep. 2022 Jun 27;12:10851. doi: 10.1038/s41598-022-14211-z (PMC9237114; doi:10.1038/s41598-022-14211-z)
Supplement: Supplementary file 1 — Supplementary Information. [file 41598_2022_14211_MOESM1_ESM.docx]

**Supplementary Information**

**Section 1:** Biochemistry parameter values available including reference ranges for feline samples.

**Section 2:** Western blot analysis confirming the recognised EV markers (uncropped blot).

**Section 3:** LC/MS-MS analysis of human and feline EVs.

**Section 4:** Asymmetrical flow field flow fractionation elution method detail.

**Section 5:** Identification of each metabolite in human and feline EVs.

**Section 1.**

**Table 1.** Biochemistry parameter values available including reference ranges for feline samples.

| **Sample ID** | **Species** | **TP_2 (g/L)** | **ALB (g/L)** | **Globulin (g/L)** | **Calcium (mmol/L)** | **UREA (mmol/L)** | **CREAT (umol/L)** | **CO2**  **(mmol/L)** |
| --- | --- | --- | --- | --- | --- | --- | --- | --- |
| Ref Range |  | 59-78 | 25-35 | 24-40 | 1.5-3.3 | 6.6-10 | 40-170 | 17-24 |
| Units |  |  |  |  |  |  |  |  |
| Feline 01 | Feline | 74.8 | 36 | 38.8 | 2.5 | 7.3 | 100 | 21 |
| Feline 02 | Feline | 70.3 | 36.2 | 34.1 | 2.5 | 18.1 | 164 | 24.9 |
| Feline 03 | Feline |  |  |  |  | 22.3 | 496 |  |
| Feline 04 | Feline | 61.4 | 31.6 | 29.8 | 2.3 | 7.2 | 68 | 19.8 |
| Feline 05 | Feline | 84.7 | 36.2 | 48.5 | 2.5 | 13.4 | 115 | 19.4 |
| Feline 06 | Feline | 72.9 | 36.9 | 36 | 2.5 | 10.5 | 125 | 25.8 |
| Feline 07 | Feline |  |  |  |  | 20.8 | 365 |  |
| Feline 08 | Feline | 73.7 | 39.4 | 34.3 | 2.7 | 30.8 | 351 | 24.4 |
| Feline 09 | Feline | 70.3 | 40.1 | 30.2 | 2.6 | 10.5 | 97 |  |
| Feline 10 | Feline | 50.4 | 29.1 | 21.3 | 2.2 | 5.6 | 73 | 17.4 |
| Feline 11 | Feline | 72.6 | 43.1 | 29.5 | 2.7 | 7.8 | 92 | 23.3 |
| Feline 12 | Feline | 71.2 | 35.9 | 35.3 | 2.4 | 9.6 | 160 | 18.9 |
| Feline 13 | Feline | 60.8 | 32.4 | 28.4 | 2.4 | 7 | 59 | 17 |
|  |  |  |  |  |  |  |  |  |
| Feline 14 | Feline | 76.5 | 38 | 38.5 | 2.6 | 6.6 | 53 | 20.1 |
| Feline 15 | Feline | 68.2 | 38.5 | 29.7 |  | 7.5 |  |  |
| Feline 16 | Feline | 78.6 | 34.9 | 43.7 | 2.4 | 11.8 | 125 | 34.2 |
| Feline 17 | Feline | 77.7 | 43.1 | 34.6 | 2.7 | 9.5 | 130 | 18.9 |
| Feline 18 | Feline | 82.6 | 33.3 | 49.3 | 2.4 | 12.3 | 115 |  |
| Feline 19 | Feline | 69.5 | 32 | 37.5 | 2.3 | 14.3 | 106 |  |
| Feline 20* | Feline |  |  |  |  |  |  |  |

| **Sample ID** | **LIPASE (U/L)** | **AMY (U/L)** | **Glucose (mmol/L)** | **CHOL (mmol/L)** | **TRIGS (mmol/L)** | **ALP (U/L)** | **GGT (U/L)** | **Total bilirublin (umol/L)** |
| --- | --- | --- | --- | --- | --- | --- | --- | --- |
| Ref Range | 0-90 | 0-1184 | 03-Jun | 1.8-6.5 | 0.09-0.68 | 0-66 | 0-73 | 1.7-5.1) |
| Units |  |  |  |  |  |  |  |  |
| Feline 01 | 19 | 1632 | 5.16 | 2.88 | 0.4 | 121 | 0 | 0.6 |
| Feline 02 | 19 | 1189 | 4.87 | 4.2 | 0.4 | 53 | 0 | 0.8 |
| Feline 03 |  |  |  |  |  |  |  |  |
| Feline 04 | 14 | 1069 | 4.36 | 3.43 | 0.3 | 48 | 1 | 2.2 |
| Feline 05 | 17 | 820 | 6.29 | 4.71 | 0.9 | 126 | 0 | 0.9 |
| Feline 06 | 23 | 1497 | 8.53 | 5.08 | 0.4 | 58 | 0 | 0 |
| Feline 07 |  |  |  |  |  |  |  |  |
| Feline 08 | 19 | 1492 | 8.32 | 5.79 | 0.4 | 25 | 1 | 66 |
| Feline 09 | 19 | 765 | 5.29 | 3.63 |  | 281 |  | 0.8 |
| Feline 10 | 13 | 361 | 7.73 | 1.84 | 0.1 | 35 | 0 | 1.1 |
| Feline 11 |  | 536 | 4.72 | 4.72 |  |  |  |  |
| Feline 12 |  | 1199 | 7.2 | 5.09 |  |  |  |  |
| Feline 13 | 22 | 997 | 5.08 | 3.48 | 0.3 | 65 | 2 | 2.7 |
|  |  |  |  |  |  |  |  |  |
| Feline 14 | 22 | 828 | 4.77 | 5.36 | 0.6 | 215 | 0 | 372 |
| Feline 15 |  |  | 5.86 | 2.77 | 0.5 | 71 | 0 | 1.5 |
| Feline 16 | 21 | 1163 | 6 | 4.15 | 0.4 | 59 | 0 | 121 |
| Feline 17 | 21 | 996 | 6.89 | 3.28 | 0.6 | 5 | 2 | 0.6 |
| Feline 18 |  |  |  |  |  |  |  |  |
| Feline 19 |  |  |  |  |  |  |  |  |
| Feline 20* |  |  |  |  |  |  |  |  |

| **Sample ID** | **ALT (U/L)** | **PHOS (mmol/L)** | **GLDH (U/L)** | **CK**  **(U/L)** | **AST (U/L)** | **ISE-Na (mmol/L)** | **ISE-K (mmol/L** | **ISE-CI (mmol/L)** |
| --- | --- | --- | --- | --- | --- | --- | --- | --- |
| Ref Range | 0-45 | 1.4-2.5 | 0-17 | 0-122 | 0-70 | 147-156 | 4 - 4.5 | 108-122 |
| Units |  |  |  |  |  |  |  |  |
| Feline 01 | 45 | 1.9 | 0 | 85 | 21 | 152 | 3.66 | 120 |
| Feline 02 | 17 | 1.5 | 0 | 128 | 11 | 154 | 4.1 | 118 |
| Feline 03 |  |  |  |  |  |  |  |  |
| Feline 04 | 42 | 1.7 | 1 | 62 | 20 | 154 | 3.88 | 125 |
| Feline 05 | 78 | 1.1 | 2 | 260 | 26 | 152 | 3.92 | 119 |
| Feline 06 | 32 | 1 | 0 | 229 | 14 | 157 | 3.44 | 121 |
| Feline 07 |  |  |  |  |  |  |  |  |
| Feline 08 | 1.8 | 2 | 2 | 184 | 28 | 146 | 3.78 | 106 |
| Feline 09 | 75 | 3.2 | 215 |  | 47 | 153 | 4.41 | 120 |
| Feline 10 | 59 | 1.9 | 10 | 465 | 102 | 155 | 4.02 | 123 |
| Feline 11 |  | 1.7 |  |  |  | 157 | 3.35 | 120 |
| Feline 12 |  | 1.3 |  |  |  | 152 | 4.79 | 121 |
| Feline 13 | 107 | 1.9 | 5 | 127 | 23 | 153 | 3.45 | 123 |
|  |  |  |  |  |  |  |  |  |
| Feline 14 | 0.7 | 2.5 | 33 | 191 | 72 | 161 | 5.36 | 125 |
| Feline 15 | 28 |  | 1 | 75 | 12 |  |  |  |
| Feline 16 | 13.9 | 1.1 | 10 | 137 | 43 | 152 | 2.64 | 109 |
| Feline 17 | 40 | 1.5 | 3 | 154 | 17 | 157 | 5.12 | 122 |
| Feline 18 | 40 | 1.2 |  | 143 | 35 | 154 | 4.25 | 118 |
| Feline 19 | 37 | 1.5 |  | 267 | 16 | 154 | 3.48 | 116 |
| Feline 20* |  |  |  |  |  |  |  |  |

**Results not available*

**Section 2.**

Western blot analysis confirming the recognised EV markers, according to the MISEV guidelines 2018 (Entire blot and corresponding Ponceau Stain – Figure 5).


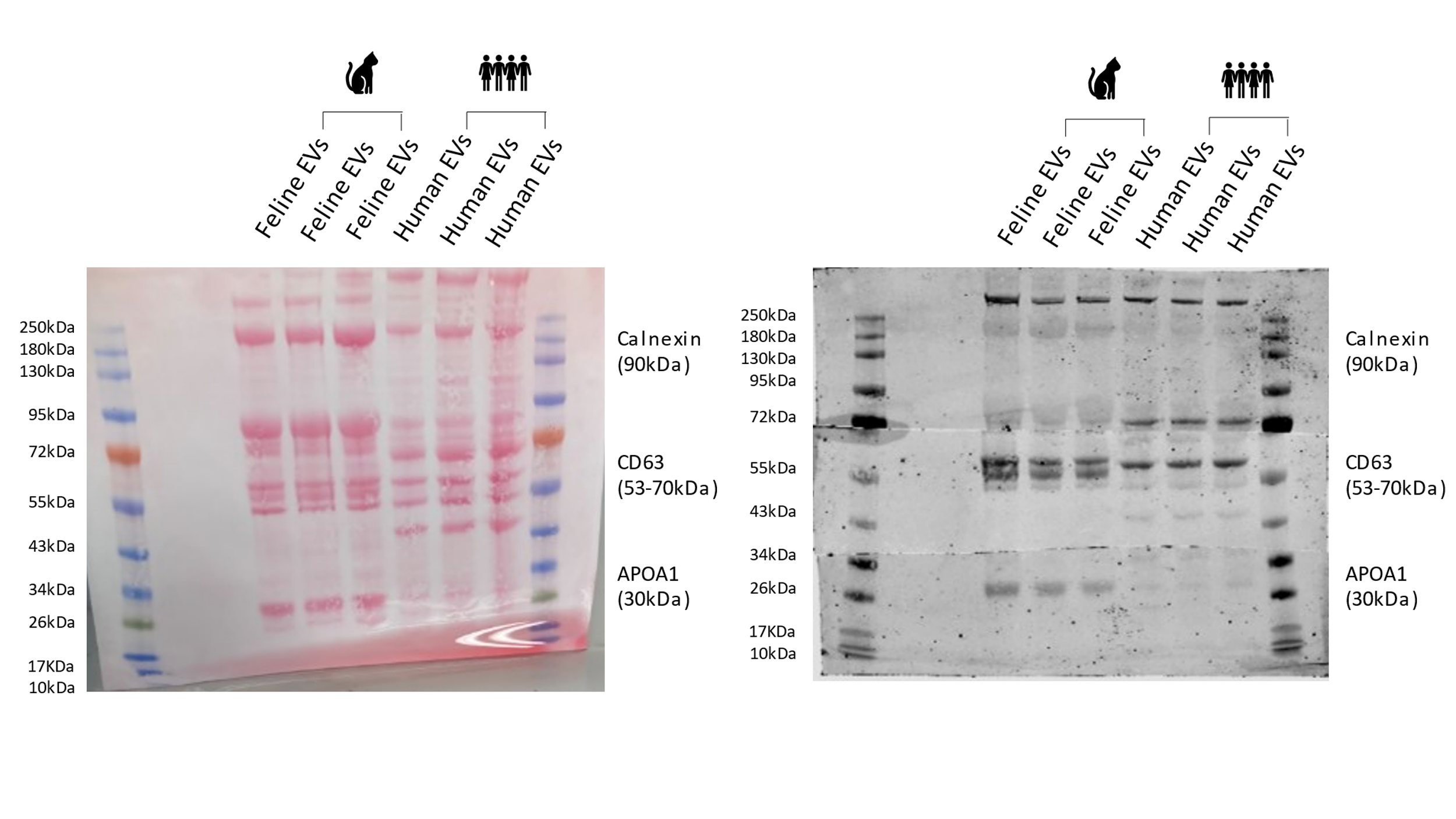


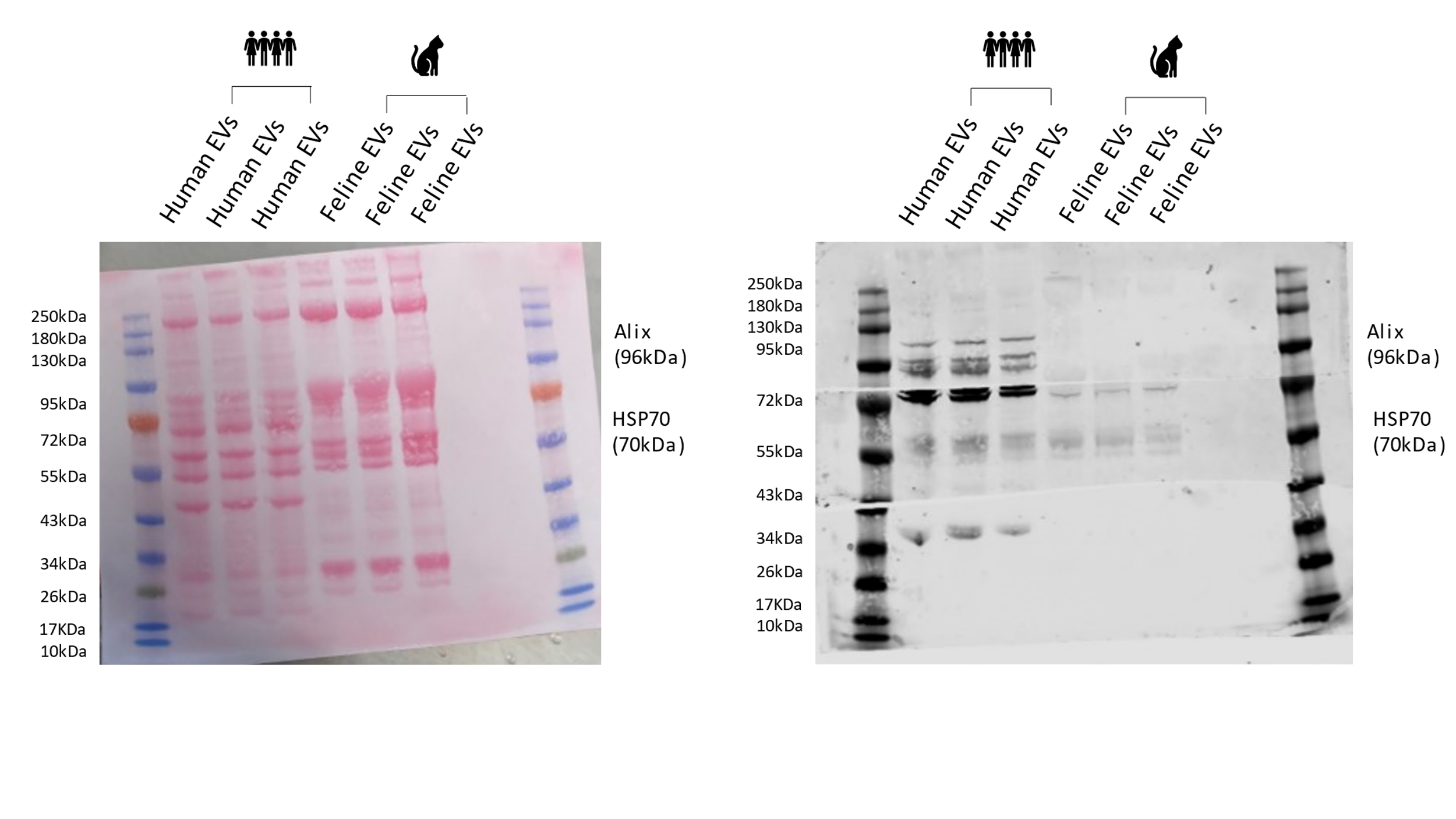


**Section 3. LC/MS-MS analysis of human and feline EVs.**

**Table 2**. LC/MS-MS was carried out on human and feline extracellular vesicles. A comparison of the proteins identified in human and feline EVs showed 55 common proteins identified in EVs from both species.

| 1. VWF |
| --- |
| 1. cd5l |
| 1. FN1 |
| 1. MYH9 |
| 1. F13A1 |
| 1. ACTN1 |
| 1. ALB |
| 1. CP |
| 1. PROS1GSNPON1 |
| 1. LOC101098159 |
| 1. TLN1 |
| 1. LOC101089505 |
| 1. FLNA |
| 1. APOA1 |
| 1. APOB |
| 1. C1QC |
| 1. ITIH2 |
| 1. FBLN1 |
| 1. C1QB |
| 1. HP |
| 1. CLU |
| 1. C1S |
| 1. PKM |
| 1. FGG |
| 1. APOA4 |
| 1. PLEK |
| 1. SERPING1 |
| 1. A2M |
| 1. C4BPA |
| 1. KRT17 |
| 1. KRT10 |
| 1. ITIH1 |
| 1. A2M |
| 1. C5 |
| 1. SLC4A1 |
| 1. ITGA2B |
| 1. FGA |
| 1. PIGR |
| 1. CFP |
| 1. LGALS1 |
| 1. VTN |
| 1. TF |
| 1. SERPINA1 |
| 1. TTR |
| 1. THBS1 |
| 1. FGB |
| 1. C4BPA |
| 1. APOA1 |
| 1. C1QA |
| 1. APOC3 |
| 1. GNB1 |
| 1. FBLN1 |
| 1. LOC101089505 |
| 1. TF |
| 1. VCL |

**Table 3.** STRING analysis of protein IDs common in both human and feline EVs was carried out to confirm the similarities in human and feline EV protein function. Protein IDs (86 IDs) were searched against the *homo sapiens* database using STRING. The top 5 biological processes (gene otology) and KEGG pathways associated with both human and feline plasma derived EVs were reported. Statistical and enrichment data was also analysed.

| **55 overlapping proteins identified in both human and feline extracellular vesicles by LC-MS/MS** | | | |
| --- | --- | --- | --- |
| **Gene Otology (Biological Processes)** | **Count in Network** | **Strength:**  **(Log10 observed/expected)** | **False Discovery Rate (p-value)** |
| Positive regulation of substrate dependent cell migration, cell attachment to substrate | 2 of 3 | 2.45 | 0.0363 |
| Negative regulation of complement activation, lectin pathway | 2 of 2 | 2.63 | 0.0025 |
| Chylomicron assembly | 4 of 10 | 2.23 | 0.000046 |
| Negative regulation of very low-density lipoprotein particle remodelling | 2 of 3 | 2.45 | 0.0041 |
| Chylomicron remodelling | 4 of 9 | 2.28 | 0.0000342 |
| **KEGG Pathways** | **Count in Network** | **Strength:**  **(Log10 observed/expected)** | **False Discovery Rate (p-value)** |
| Complement and coagulation cascades | 16 of 82 | 1.92 | 1 x 10^-23^ |
| Vitamin digestion and absorption | 3 of 24 | 1.73 | 0.0013 |
| Staphlococcus aureus infection | 8 of 86 | 1.6 | 7.76 x 10^-9^ |
| Pertussis | 7 of 74 | 1.6 | 8.28 x 10^-8^ |
| Cholesterol Metabolism | 4 of 48 | 1.55 | 0.00028 |

**Section 4.**

Elution Method

Detector Flow: 0.25 ml/min

Smart Stream Splitting: 0.25 ml/min

Delay Time: 5 min (Stabilisation of all flow rates before injection)

Injection and Focus Time: 15 min

Injection Flow: 0.20 ml/min

Focus Flow: 1.30 ml/min

Cross Flow: 1.0 ml/min

Transition Time: 0.5 min

20 min Cross Flow 1.0 ml/min to 0.22 ml/min linear

5 min Cross Flow 0.22 ml/min to 0.1 ml/min power 0.8

2 min Cross Flow 0.1 ml/min to 0.08 ml/min power 0.7

6 min Cross Flow 0.08 ml/min to 0.035 ml/min power 0.8

35 min Cross Flow constant


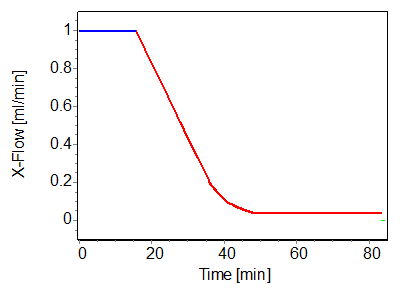


**Section 5.**

**Table 4.** Identification of each metabolite in human and feline EVs.

| Metabolites | Human | Feline |
| --- | --- | --- |
| lysoPC a C14:0 | X ^(a)^ | X |
| lysoPC a C16:0 | 🗸 | 🗸^(b)^ |
| lysoPC a C16:1 | 🗸 | X |
| lysoPC a C17:0 | 🗸 | 🗸 |
| lysoPC a C18:0 | 🗸 | 🗸 |
| lysoPC a C18:1 | 🗸 | 🗸 |
| lysoPC a C18:2 | 🗸 | 🗸 |
| lysoPC a C20:3 | 🗸 | 🗸 |
| lysoPC a C20:4 | 🗸 | 🗸 |
| lysoPC a C24:0 | X | X |
| lysoPC a C26:0 | 🗸 | X |
| lysoPC a C26:1 | X | X |
| lysoPC a C28:0 | 🗸 | 🗸 |
| lysoPC a C28:1 | 🗸 | 🗸 |
| PC aa C24:0 | 🗸 | 🗸 |
| PC aa C26:0 | 🗸 | X |
| PC aa C28:1 | 🗸 | X |
| PC aa C30:0 | 🗸 | 🗸 |
| PC aa C30:2 | 🗸 | 🗸 |
| PC aa C32:0 | 🗸 | 🗸 |
| PC aa C32:1 | 🗸 | 🗸 |
| PC aa C32:2 | 🗸 | 🗸 |
| PC aa C32:3 | 🗸 | 🗸 |
| PC aa C34:1 | 🗸 | 🗸 |
| PC aa C34:2 | 🗸 | 🗸 |
| PC aa C34:3 | 🗸 | 🗸 |
| PC aa C34:4 | 🗸 | X |
| PC aa C36:0 | X | X |
| PC aa C36:1 | 🗸 | 🗸 |
| PC aa C36:2 | 🗸 | 🗸 |
| PC aa C36:3 | 🗸 | 🗸 |
| PC aa C36:4 | 🗸 | 🗸 |
| PC aa C36:5 | 🗸 | 🗸 |
| PC aa C36:6 | 🗸 | X |
| PC aa C38:0 | X | X |
| PC aa C38:1 | 🗸 | 🗸 |
| PC aa C38:3 | 🗸 | 🗸 |
| PC aa C38:4 | 🗸 | 🗸 |
| PC aa C38:5 | 🗸 | 🗸 |
| PC aa C38:6 | 🗸 | 🗸 |
| PC aa C40:1 | X | X |
| PC aa C40:2 | 🗸 | 🗸 |
| PC aa C40:3 | 🗸 | 🗸 |
| PC aa C40:4 | 🗸 | 🗸 |
| PC aa C40:5 | 🗸 | 🗸 |
| PC aa C40:6 | 🗸 | 🗸 |
| PC aa C42:0 | X | X |
| PC aa C42:1 | X | X |
| PC aa C42:2 | X | X |
| PC aa C42:4 | X | X |
| PC aa C42:5 | 🗸 | X |
| PC aa C42:6 | 🗸 | X |
| PC ae C30:0 | 🗸 | X |
| PC ae C30:1 | 🗸 | X |
| PC ae C30:2 | 🗸 | 🗸 |
| PC ae C32:1 | 🗸 | 🗸 |
| PC ae C32:2 | 🗸 | 🗸 |
| PC ae C34:0 | 🗸 | 🗸 |
| PC ae C34:1 | 🗸 | 🗸 |
| PC ae C34:2 | 🗸 | 🗸 |
| PC ae C34:3 | 🗸 | 🗸 |
| PC ae C36:0 | 🗸 | 🗸 |
| PC ae C36:1 | 🗸 | 🗸 |
| PC ae C36:2 | 🗸 | 🗸 |
| PC ae C36:3 | 🗸 | 🗸 |
| PC ae C36:4 | 🗸 | 🗸 |
| PC ae C36:5 | 🗸 | 🗸 |
| PC ae C38:0 | 🗸 | X |
| PC ae C38:1 | X | X |
| PC ae C38:2 | 🗸 | 🗸 |
| PC ae C38:3 | 🗸 | 🗸 |
| PC ae C38:4 | 🗸 | 🗸 |
| PC ae C38:5 | 🗸 | 🗸 |
| PC ae C38:6 | 🗸 | 🗸 |
| PC ae C40:1 | 🗸 | X |
| PC ae C40:2 | 🗸 | 🗸 |
| PC ae C40:3 | 🗸 | 🗸 |
| PC ae C40:4 | 🗸 | 🗸 |
| PC ae C40:5 | 🗸 | 🗸 |
| PC ae C40:6 | 🗸 | 🗸 |
| PC ae C42:0 | X | X |
| PC ae C42:1 | X | X |
| PC ae C42:2 | 🗸 | 🗸 |
| PC ae C42:3 | 🗸 | X |
| PC ae C42:4 | X | X |
| PC ae C42:5 | X | X |
| PC ae C44:3 | 🗸 | X |
| PC ae C44:4 | X | X |
| PC ae C44:5 | 🗸 | X |
| PC ae C44:6 | 🗸 | X |
| SM (OH) C14:1 | 🗸 | 🗸 |
| SM (OH) C16:1 | 🗸 | 🗸 |
| SM (OH) C22:1 | 🗸 | 🗸 |
| SM (OH) C22:2 | 🗸 | 🗸 |
| SM (OH) C24:1 | 🗸 | 🗸 |
| SM C16:0 | 🗸 | 🗸 |
| SM C16:1 | 🗸 | 🗸 |
| SM C18:0 | 🗸 | 🗸 |
| SM C18:1 | 🗸 | 🗸 |
| SM C20:2 | 🗸 | 🗸 |
| SM C22:3 | X | X |
| SM C24:0 | 🗸 | 🗸 |
| SM C24:1 | 🗸 | 🗸 |
| SM C26:0 | 🗸 | X |
| SM C26:1 | 🗸 | 🗸 |
| Hexose | 🗸 | 🗸 |
| Alanine | X | X |
| Arginine | X | X |
| Asparagine | X | X |
| Aspartate | X | X |
| Citrulline | X | X |
| Glutamine | X | X |
| Glutamate | X | X |
| Glycine | X | X |
| Histidine | X | X |
| Isoleucine | X | X |
| Leucine | X | X |
| Lysine | X | X |
| Methionine | X | X |
| Ornithine | X | X |
| Phenylalanine | X | X |
| Proline | X | X |
| Serine | X | X |
| Threonine | X | X |
| Tryptophan | X | X |
| Tyrosine | X | X |
| Valine | X | X |
| Acetylornithine | X | X |
| Asymmetric dimethylarginine | X | X |
| α-Aminoadipic acid | X | X |
| cis-4-Hydroxyproline | X | X |
| Carnosine | X | X |
| Creatinine | X | X |
| Dihydroxyphenylalanine | X | X |
| Dopamine | X | X |
| Histamine | X | X |
| Kynurenine | X | X |
| Methionine sulfoxide | X | X |
| Nitrotyrosine | X | X |
| Phenylethylamine | X | X |
| Putrescine | 🗸 | X |
| Sarcosine | X | X |
| Symmetric dimethylarginine | X | X |
| Serotonin | X | X |
| Spermidine | 🗸 | 🗸 |
| Spermine | 🗸 | 🗸 |
| trans-4-Hydroxyproline | X | X |
| Taurine | 🗸 | X |

1. X: < limit of detection. (b) 🗸: > limit of detection.

lysoPCs: Lysophosphatidylcholines; PCs: Phosphatidylcholines; SMs: Sphingomyelins.
